# Impacts of road networks on the geography of floristic collections in China

**DOI:** 10.1016/j.pld.2025.02.001

**Published:** 2025-02-21

**Authors:** Jingyang He, Wenjing Yang, Qinghui You, Qiwu Hu, Mingyang Cong, Chao Tian, Keping Ma

**Affiliations:** a Key Laboratory of Poyang Lake Wetland and Watershed Research (Jiangxi Normal University), Ministry of Education, Nanchang 330022, China; b School of Geography and Environment, Jiangxi Normal University, Nanchang 330022, China; c Key Laboratory of Biodiversity Conservation and Bioresource Utilization of Jiangxi Province, College of Life Sciences, Jiangxi Normal University, Nanchang 330022, China; d State Key Laboratory of Vegetation and Environmental Change, Institute of Botany, Chinese Academy of Sciences, 20 Nanxincun, Xiangshan, Beijing 100093, China

**Keywords:** Biological sampling, Environmental and socio-economic variables, Plants, Road-map effect, Species occurrence records

## Abstract

Biological collections are critical for the understanding of species distributions and for formulating biodiversity conservation strategies. However, biological collections are susceptible to various biases, including the “road-map effect”, meaning that the geography of biological collections can be influenced by road networks. Here, using species occurrence records derived from 921,233 plant specimens, we quantified the intensity of the “road-map effect” on floristic collections of China, and investigated its relationships with various environmental and socio-economic variables. Species occurrence records mainly distributed in major mountain ranges, while lowlands were underrepresented. The distance of species occurrence records to the nearest road decreased from 19.54 km in 1960s to 3.58 km in 2010s. These records showed significant clustering within 5 km and 10 km buffer zones of roads. The road density surrounding these records was significantly higher than that in random patterns. Collectively, our results confirmed a significant “road-map effect” in the floristic collections of China, and this effect has substantially intensified from the 1960s to the 2010s, even after controlling for the impact of road network expansion. Topographic, climatic and socio-economic variables that determine regional species diversity, vegetation cover and human impact on vegetation played crucial roles in predicting the intensity of the “road-map effect”. Our findings indicate that biological surveys have become increasingly dependent on road networks, a trend rarely reported in published studies. Future floristic surveys in China should prioritize the lowland areas that have experienced stronger human disturbances, as well as remote areas that may harbor more unique and rare species.

## Highlights

• Floristic collections in China are mainly concentrated in major mountain ranges, while lowlands are underrepresented.
• Floristic surveys have become increasingly dependent on road networks from the 1960s to the 2010s.
• Regional species diversity, vegetation cover, and human disturbances affect the intensity of “road-map effect”.

## 1 Introduction

Biological collections are a fundamental resource for understanding the temporal and spatial distributions of species in the natural world (Nualart et al., 2017). Since the 14th century, numerous specimens have been collected by scientists and explorers and systematically deposited in museums and herbaria (Bordelon, 2022). Over the past two decades, a portion of these biological collections has been digitized, with their information now accessible through online databases such as the Global Biodiversity Information Facility (Canhos et al., 2004). The accessibility of vast species occurrence data has greatly accelerated the advancement of disciplines related to biodiversity and biogeography (Beck et al., 2013; Zizka et al., 2021).

Nevertheless, species occurrence data are prone to various biases, especially geographical bias (Bowler et al., 2022; Zhang et al., 2024). A number of studies have demonstrated that currently available species occurrence data are not evenly distributed across regions and are heavily influenced by environmental and socio-economic factors (Meyer et al., 2015; Cosentino and Maiorano, 2021). For instance, regions of Ecuador characterized by higher temperatures, lower precipitation, and pronounced seasonality tended to be underrepresented in species occurrence datasets (Loiselle et al., 2008). This is often because these regions typically exhibit lower species diversity, while collectors tend to more often visit environments that are comparatively richer in species. The tropical Andes is one of the most species-rich regions in the world. However, species occurrence records in the northern Andes, particularly in Venezuela and Colombia, remain relatively sparse due to historical internal conflicts, although the situation has been improved in recent years (Vargas et al., 2022). This conflict has made fieldwork extremely risky, leading to minimal collecting efforts in these regions. In addition, the level of economic development also determines the availability of species occurrence records in a region. For instance, more economically developed countries in Africa generally have a greater amount of species occurrence data compared to those developing countries (Reddy and Dávalos, 2003).

An especially prevalent sampling bias is the so-called “road-map effect” (Crisp et al., 2001), which describes how the geographical distribution of biological collections is influenced by road networks (Lituma and Buehler, 2016). For instance, strong correlations have been observed between the density of access routes and the number of species occurrence records across various taxonomic groups in Brazil (Oliveira et al., 2016). A majority of the species occurrence records are concentrated within the 1 km buffer zone of these access routes, known as the roadside bias. Similar patterns have been observed in arid inland regions of Australia, where plant collections are predominantly found near a few main roads (Chapman and Busby, 1994). In Norway, the density of species occurrence records is much lower in remote areas with limited accessibility compared to highly accessible areas (Petersen et al., 2021).

According to published studies, there are generally three different methods to quantify the “road-map effect”. The first method measures the distance of species occurrence records to the nearest roads (Parnell et al., 2003). If the number of species occurrence records decreases with increasing distance from the roads, it suggests that there is a significant “road-map effect” in the species occurrence datasets. Another method is to quantify the probability of biological collections falling within a certain buffer zone (usually 5 km) of roads (Zizka et al., 2021). If there is a higher likelihood of species records falling within a given buffer zone when compared to a random distribution, the roadside bias is considered to be statistically significant (Kadmon et al., 2004). The third method involves assessing the accessibility of areas where species occurrence records are located, typically quantified by the road density within the surveyed areas (Yang et al., 2014). Increased collecting efforts in areas with higher accessibility suggest a notable “road-map effect” in biological collections. However, very few studies have simultaneously employed these three methods to quantify the intensity of the “road-map effect” in species occurrence datasets, although each of these methods reveals the influence of road networks on the spatial distribution of species occurrence records from distinct perspectives.

The intensity of the “road-map effect” can vary largely across different regions, being influenced by a combination of various factors including climatic, topographic, and socio-economic variables. For instance, plant occurrence records are concentrated in the wet and mountainous areas of Israel despite the road density being relatively low (Kadmon et al., 2004). In contrast, mammal occurrence records on the island of Borneo display a distinct bias toward urban areas with high human population and road density, attributed to enhanced accommodation and transportation infrastructure (Zizka et al., 2021). It has also been found that collectors tend to stick closer to roads in the areas with low species richness (e.g., desert areas), owing to the limited opportunities for collecting additional species (Crisp et al., 2001). Thus, exploring the influence of various environmental and socio-economic variables on the “road-map effect” would be valuable for predicting the impact of road networks on the distribution of species occurrence records, and facilitating the mitigation of this impact during data utilization.

China is one of the most biodiverse countries in the world, with over 30,000 native species of vascular plants (Zhuang et al., 2021). The flora of China have been extensively surveyed over the past centuries, and more than 10 million specimens have been collected and deposited in herbaria (Liu et al., 2022b). These collections have served as crucial foundations for understanding the geographical distribution of plant species and formulating biodiversity conservation policies in China (Peng et al., 2021). Yet, floristic collections in China suffer strong geographical bias, and only 9% of all counties have been completely sampled (Yang et al., 2013). The rapid economic development of China in recent decades has led to a substantial expansion of its road networks (Pan et al., 2024), greatly facilitating field surveys and biological collections. However, this expansion could exacerbate the impact of road networks on biological surveys, potentially increasing the dependency of such surveys on roads (Grilo et al., 2010).

In this paper, we utilize ∼0.92 million species occurrence records derived from vascular plant specimens with precise collection location information and collected from 1960 to 2020. Our objectives are threefold: (1) to quantify the intensity of the “road-map effect” in the floristic collections of China; (2) to assess whether the intensity of the “road-map effect” varies across different time periods; and (3) to identify the primary environmental and socio-economic factors influencing the effect of road networks on the geography of floristic collections in China.

## 2 Materials and methods

### 2.1 Species occurrence data

Species occurrence records, derived from 8,137,097 vascular plant specimens, were obtained from the Chinese Virtual Herbarium (CVH, https://www.cvh.ac.cn/). All data were quality-controlled according to the following criteria: (1) duplicate records for the same specimen that may have occurred during the digitization process were removed; (2) only records containing precise information on collection locations and years were retained; (3) records were georeferenced to the most detailed geographical level available, down to the village level and finer granularities. These criteria yielded 921,233 records collected from 1960 to 2020, representing 27,838 species, which were used for subsequent analyses.

### 2.2 Road map data

The mileage of roads in China from 1960 to 2020 was obtained from datasets published by the National Bureau of Statistics of China (Department of Comprehensive Statistics of National Bureau of Statistics, 2010; National Bureau of Statisitics of China, 2009–2020). Each occurrence record was associated with the road network of the corresponding time period. Road maps of China for the years 1962, 1974, and 1986 were digitally converted from published paper maps using ArcGIS 10.2 (China Cartographic Publishing House, 1962, 1974, 1986), while those for the years 1995, 2000 and 2012 were downloaded from the Resource and Environmental Science Data Platform (https://www.resdc.cn/) and the Geographic Data Platform (https://geodata.pku.edu.cn/). These datasets were utilized to represent the road networks of their respective decades. We have not obtained road map data prior to 1960; therefore, data preceding this period were excluded from the analysis.

### 2.3 Environmental and socio-economic data

Ten socio-economic variables taken over 1960 to 2020 were compiled to investigate the impacts of these factors on “road map effect” intensity, including mean elevation, elevation range, annual mean temperature, mean temperature in the coldest month of the year, potential evapotranspiration, annual precipitation, seasonality of precipitation, Normalized Difference Vegetation Index (NDVI), human population density, and gross domestic product (GDP). NDVI data was only available for the period from 1981 to 2020, whereas human population density and GDP data were available from 1995 to 2020. For analyses conducted prior to these specified periods, the nearest available data were utilized. For example, NDVI data for 1981 were used for analyses involving species occurrence data collected in the 1960s and 1970s. The mean elevation and elevation range within a specified buffer zone (5–20 km) of species occurrence records or within the county where species occurrence records were located, were employed as indicators of accessibility and topographic complexity (Rahbek and Graves, 2001). Climatic variables determine plant species diversity within an area (Qian and Kissling, 2010; Yang et al., 2013), whereas NDVI measures the density of green vegetation cover (Liu et al., 2022a), potentially influencing the appeal of the area to collectors, i.e., areas with higher species richness and superior vegetation cover are likely to be more attractive to collectors (Loiselle et al., 2008). Preferred collection areas can also be influenced by human population density and GDP; higher levels of these factors are likely to provide better infrastructure for field surveys (Reddy and Dávalos, 2003).

Temperature and precipitation data were obtained from WorldClim with a spatial resolution of 30 arc-seconds (https://worldclim.org/data/worldclim21.html). Mean elevation and elevation range were calculated from the GTOPO-30 digital elevation model at a spatial resolution of 30 arc-seconds (U.S. Geological Survey, 1996). All other datasets were sourced from the Resource and Environmental Science Data Platform (https://www.resdc.cn/), with a spatial resolution of 1 km, except for NDVI, which has a resolution of 8 km.

### 2.4 Quantification of the “road-map effect”

Three different methods were used to quantify the “road-map effect” on plant occurrence data of China. The first method calculated the distance of an occurrence record to the nearest road to assess whether the observed geographical patterns of occurrence record distributions significantly differed from random patterns. An equal number of random points, corresponding to the number of species occurrence records, were generated and distributed randomly across China. The mean distance of random points to the nearest road in each pattern was then calculated. This process was repeated 1000 times, and the 5th percentile of the mean distances was computed. If the observed mean distance to road is smaller than the 5th percentile value, it suggests a significant “road-map effect”. The ratio between the observed mean distance and the mean distance derived from random patterns was calculated to assess the extent to which the observed distance deviates from random patterns. A ratio value closer to 0 indicates a greater deviation from randomness and a stronger “road-map effect”.

In addition, gamma distributions were applied to model the observed distance data. The shape parameter *k* in the probability density function of the gamma distribution determines the right skewness of the density curve, employed to indicate the rate at which occurrence record numbers decrease with distance from the road. A higher *k* value indicates a slower decay rate of occurrence record numbers with distance from the road, suggesting a weaker bias to roadsides (Husak et al., 2007). The value of *k* was estimated using the “fitdistr” function from the R package “*MASS*” (Venables and Ripley, 2002), employing maximum likelihood estimation with the scale parameter *θ* unconstrained (Appendix A).

The second method assessed whether species occurrence records showed significant clustering along roadsides, specifically within designated buffer zones of either 5 or 10 km from the roads. The distances of 5 and 10 km were selected because they can be covered on foot in 1–2 h. Each record could be classified into two categories—either within the buffer zones or outside of them. This classification enables the use of the binomial distribution to evaluate both the significance and degree of clustering of species occurrence records within these buffer zones. The evaluation is grounded in the assumption that all records are uniformly and randomly distributed across the region of interest.

A metric, denoted as *P*_*clustering*_, was developed to indicate the degree of clustering of records within the buffer zones. For regions that have more records falling within the buffer zones than expected, the value of *P*_*clustering*_ was determined through the application of the following formula:

(1) $$P_{clustering}=P\left(X\leq n_{d}-1\right)=\sum_{i=0}^{n_{d}-1}\left(\begin{array}{l} N\\ i \end{array}\right){p_{d}}^{i}{\left(1-p_{d}\right)}^{N-i}$$

where the random variable *X* follows a binomial distribution with parameters *N* and *p*_*d*_. *N* denotes the total number of species occurrence records in the relevant region, while *p*_*d*_ represents the probability for a given occurrence record falling within a given distance (*d*, either 5 or 10 km) to the nearest road. The value of *p*_*d*_ is determined as the ratio of the buffer zone area to the total area of the region. The variable *n*_*d*_ indicates the observed number of species occurrence records falling within the buffer zones. The term *P*(*X* ≤ *n*_*d*_–1) represents the cumulative probability of observing *n*_*d*_–1 or fewer records within the buffer zones. Accordingly, 1–*P*(*X* ≤ *n*_*d*_–1) corresponds to the *p*-value for the observation of *n*_*d*_ records or more within the buffer zones in a right-tailed test. If the value of *P*(*X* ≤ *n*_*d*_–1) exceeds 0.95, it suggests a significant clustering of records within the buffer zones.

For a region where the number of records within the road buffer zones is equal to the expected number (*N* × *p*_*d*_), *P*_*clustering*_ was assigned a value of 0.50. If the number of records within the buffer zones is less than the expected count, the following equation was employed to compute the *P*_*clustering*_:

(2) $$P_{clustering}=P\left(X\leq n_{d}\right)=\sum_{i=0}^{n_{d}}\left(\begin{array}{l} N\\ i \end{array}\right){p_{d}}^{i}{\left(1-p_{d}\right)}^{N-i}$$

where *P*(*X* ≤ *n*_*d*_) represents the cumulative probability of observing *n*_*d*_ or fewer records within the buffer zones. The *P*(*X* ≤ *n*_*d*_) value corresponds to the *p*-value for observing *n*_*d*_ or less records within the buffer zones in a left-tailed test. If the value of *P*(*X* ≤ *n*_*d*_) is less than 0.05, it suggests that the records are significantly situated away from road buffer zones.

The value of *P*_*clustering*_ derived from the above equations varied between 0 and 1 (excluding 0 or 1), with higher values indicating a greater degree of record clustering within the buffer zones. The *P*_*clustering*_ value was calculated at two different spatial scales: the national level and the county level. Counties are political units characterized by relatively low environmental and socio-economic heterogeneity. To obtain the *p*_*d*_ values for both national and county levels, buffer zones were created around all roads within the country, and the buffer zone area within each county was measured. The *p*_*d*_ value at the national level was calculated as the ratio of the total buffer zone area across the entire country to the country's overall area. The *p*_*d*_ value for each individual county was determined as the ratio of the buffer zone area within that county to the county's total area.

The third method calculated the road density within designated buffer zones (5 km, 10 km, 20 km, 30 km, 50 km and 60 km, respectively) of each species occurrence record. If the mean road density around the observed occurrence records is higher than that of randomly distributed patterns across the country in 1000 simulations, and it surpasses 95th percentile of the mean road density in those random patterns, it implies that sampling sites with greater accessibility tend to be sampled more frequently (Reddy and Dávalos, 2003).

### 2.5 Random forest modeling

Random forest models were employed to quantify the effects of various socio-economic and environmental variables on the intensity of “road-map effect” via all three methods. Random forests are a powerful ensemble learning method that combines the strengths of multiple decision trees to create a robust model. One of the key advantages of random forests is their ability to handle complex relationships between predictor variables and response variables, as well as to mitigate overfitting (Valavi et al., 2021). The Akaike information criterion (AIC) was used along with a step-wise backward selection strategy to choose the most parsimonious multi-predictor model (Johnson and Omland, 2004). The random forest regressions provided a measure of variance explained that is equivalent to *R*^2^ in linear regressions. The relative importance of each predictor variable in the model was calculated by randomly permuting the values of each predictor and observing the resulting change in mean square error from the original variance explained by the model (Van Buskirk and Jansen van Rensburg, 2020). In addition, Pearson correlation coefficients were computed to examine the positive or negative relationships between the response and explanatory variables.

Spatial autocorrelation in response variables and residuals of the random forest models is relatively weak, yet still statistically significant, particularly within distances of less than 300 km, as evidenced by the spatial correlograms and global Moran's *I*-tests (Fig. S1). This can lead to the overestimation of degrees of freedom and consequently an inflation of Type I errors in the random forest models (Diniz-Filho et al., 2003). Eigenfunction-based methods are effective in handling complex spatial patterns and are commonly utilized to account for spatial autocorrelation in regression analyses (Diniz-Filho and Bini, 2005). The pairwise Euclidean distances among species occurrence records involved in the random forest modeling were computed from their geographical coordinates in the Albers projection. The distance matrix was truncated at a distance of 300 km. Distances exceeding 300 km were substituted with 1200 km (four times the truncation distance), while distances of 300 km or less were preserved as originally calculated (Yang et al., 2014). This truncation distance is important because it gives more weight to short-distance effects after the filtering process. A distance of 300 km was chosen due to the observation of minimal spatial autocorrelation in residuals of non-spatial random forest models beyond this threshold (Fig. S1).

Principal coordinates of neighbor matrices (PCNM) were employed to decompose the spatial structures among species occurrence records using the “pcnm” function in the R package “*vegan*” (Borcard and Legendre, 2002). Forty eigenvectors with positive eigenvalues were derived to capture the spatial relationships among species occurrence records across various scales. Eigenvector-based spatial filters that contributed the most to the variance explained by the random forest model were subsequently included in the model until spatial autocorrelation in model residuals became insignificant. Random forest modeling was carried out using the “randomForest” function from the R package “*randomForest*” (van der Maaten et al., 2017; Appendix A).

## 3 Results

### 3.1 Road network in China

**Fig. 1:**
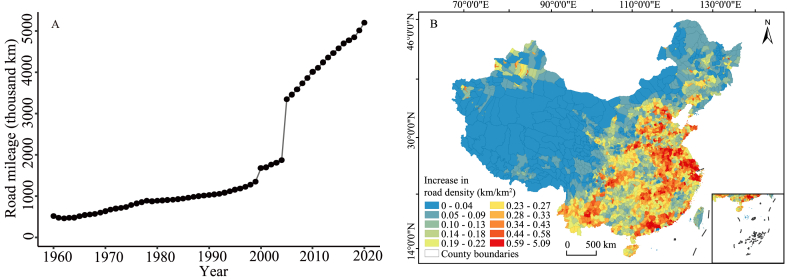
(A) Annual road mileage in China and (B) increase in road density across various counties from 1960 to 2020. (B) The legend uses a quantile classification; the map is an Albers projection. The inset in the bottom right corner show the south boundary of China, including all islands in the South China Sea.

China has dramatically increased its road network from a mileage of 519,500 km in 1960 to 5,198,100 km in 2020 (Fig. 1A). This growth was relatively slow before 2000, increasing at an average of 21,388 km per year. The growth accelerated to an average of 124,364 km per year after 2005. In general, road density tends to be higher in the eastern and southern regions compared to the western and northern regions (Fig. S2). A similar geographical pattern has been observed for the increase in road density over the last decades (Fig. 1B).

### 3.2 Species occurrence records

**Fig. 2:**
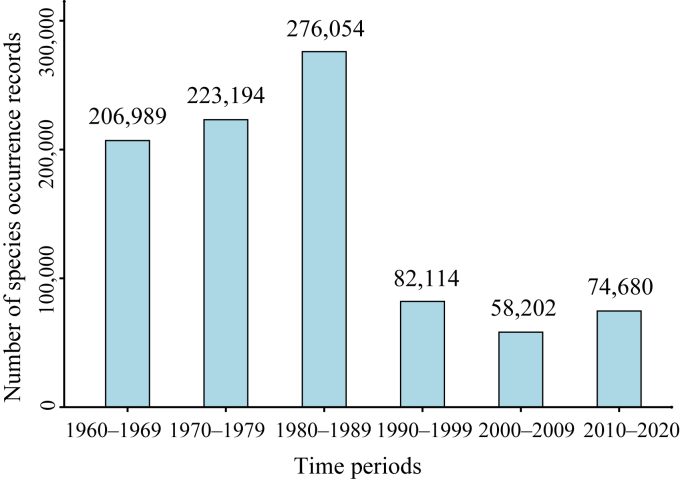
The number of species occurrence records used in this study across different time periods.

**Fig. 3:**
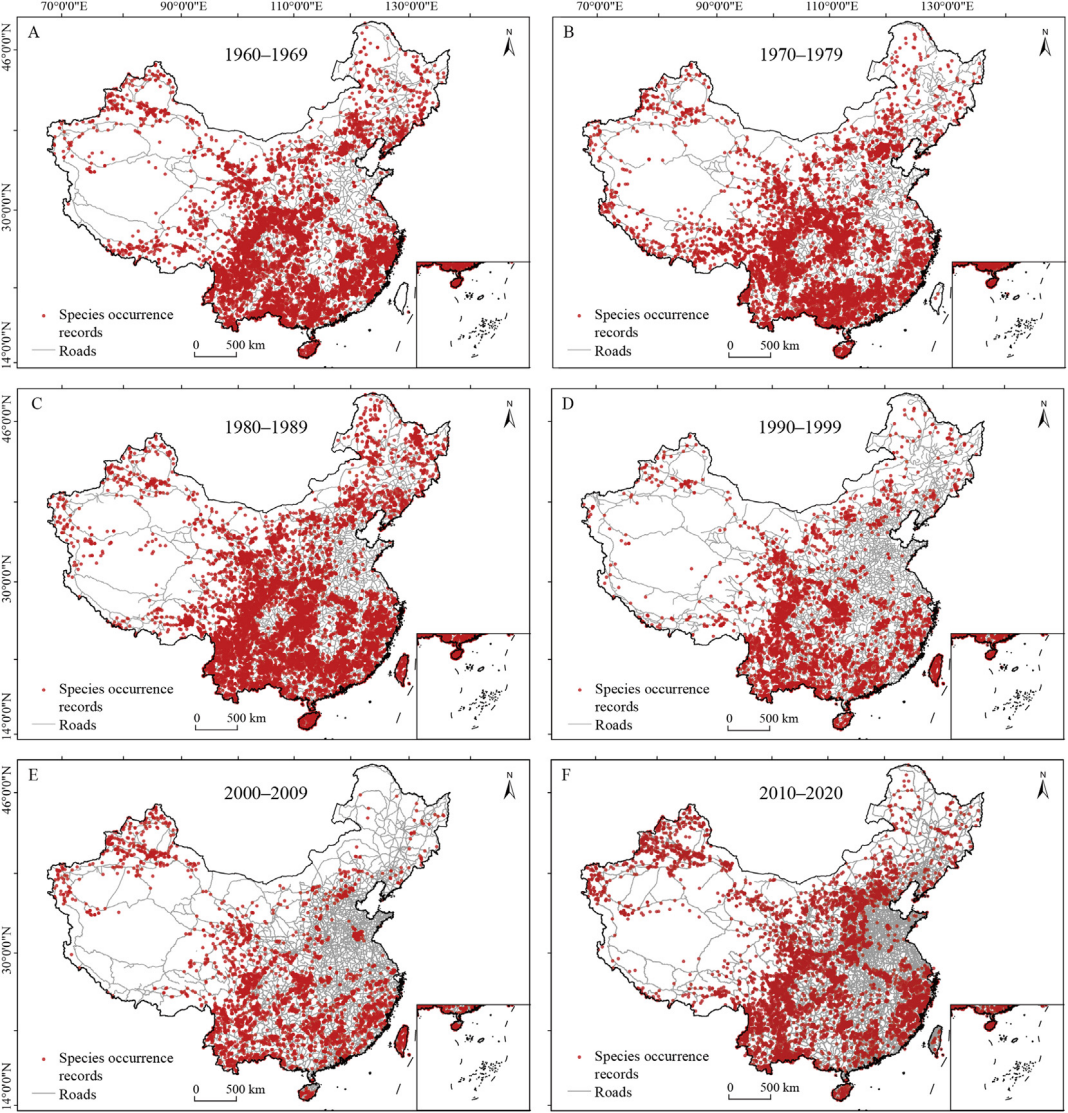
Geographical patterns of species occurrence records used in this study across different time periods. The road networks for each of the time periods are based on data from 1962, 1974, 1986, 1995, 2000, and 2012, respectively.

The number of species occurrence records that can be georeferenced to the village level and even finer granularities, and that include collection year information, varied substantially across different time periods (Fig. 2). The number of species occurrence records was highest in the 1980s, followed by the 1970s and 1960s, at 276,054, 223,194, and 206,989, respectively. By contrast, the number of species occurrence records was much fewer in the 1990s, 2000s and 2010s (with the 2010s referring to the period from 2010 to 2020), at 82,114, 58,202 and 74,680, respectively. The distribution of species occurrence records showed similar geographical patterns in different time periods (Fig. 3). These records were predominantly concentrated in southern and southwestern China, especially along the major mountain ranges (e.g., Hengduan, Daba and Nanling Mountains). In contrast, they were notably fewer in the northern (e.g., North China Plain) and northwestern regions.

### 3.3 The intensity of the “road-map effect”

**Fig. 4:**
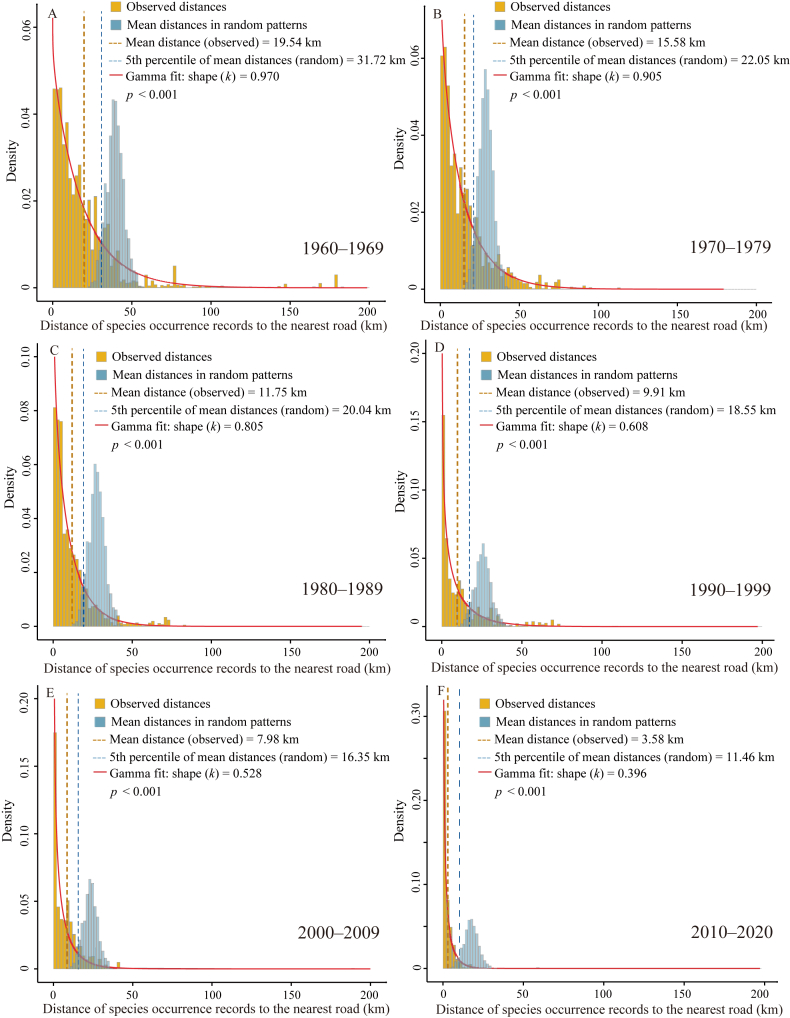
The histograms illustrate the distances of species occurrence records to the nearest road (in yellow) in different time periods, compared to the mean distances derived from randomly generated patterns (in blue). These patterns are created by randomly and uniformly distributing the same number of points as the species occurrence records across China, and the mean distance from these random points to the nearest road is calculated for each pattern. The process is iterated for 1000 times, resulting in 1000 mean distance values. The red curves represent the gamma fit applied to the observed distance values, with the shape parameter (*k*) indicating the rate at which the number of species occurrence records decreases as the distance to the road increases. The yellow and blue dashed vertical lines represent the observed mean distance of species occurrence records to the nearest road and the 5th percentile value of the mean distances in the random patterns, respectively.

**Fig. 5:**
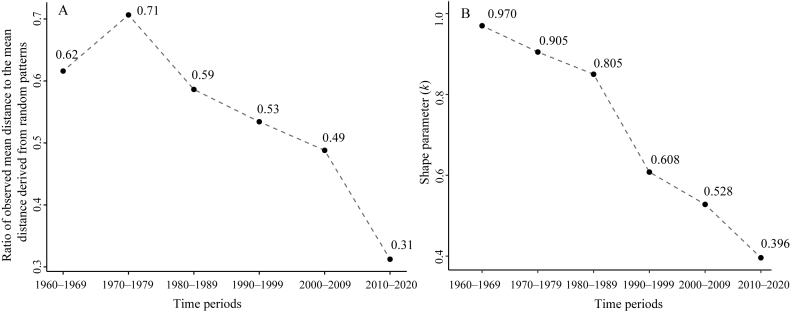
Trends in the ratios between the observed mean distance of species occurrence records to the nearest road and the mean distance derived from random patterns (A), and the shape parameter (*k*) of gamma fits for the observed distances (B) from the 1960s to the 2010s (including 2020).

The number of species occurrence records decreased as the distance from roads increased in all time periods (Fig. 4). The mean distance of species occurrence records to the nearest road decreased from 19.54 km in the 1960s to 3.58 km in the 2010s, significantly lower that of random patterns in the corresponding time period (*p* < 0.001). The ratio of observed mean distance to the mean distance derived from random patterns, decreased from 0.62 in the 1960s to 0.31 in the 2010s (Fig. 5A), suggesting that the distribution of species occurrence records has increasingly deviated from random patterns. The shape parameter (*k*) in the probability density function of the gamma distribution fitted to the observed distance data declined from 0.97 in the 1960s to 0.40 in the 2010s (Fig. 5B), suggesting that the rate at which record numbers decreased with increasing distance from roads accelerated from the 1960s to the 2010s.

**Fig. 6:**
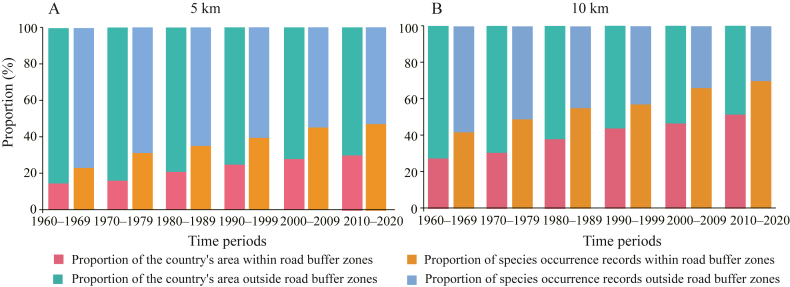
Proportions of the country's area and species occurrence records located within and outside the 5 km and 10 km buffer zones of roads at the national scale in different time periods.

**Fig. 7:**
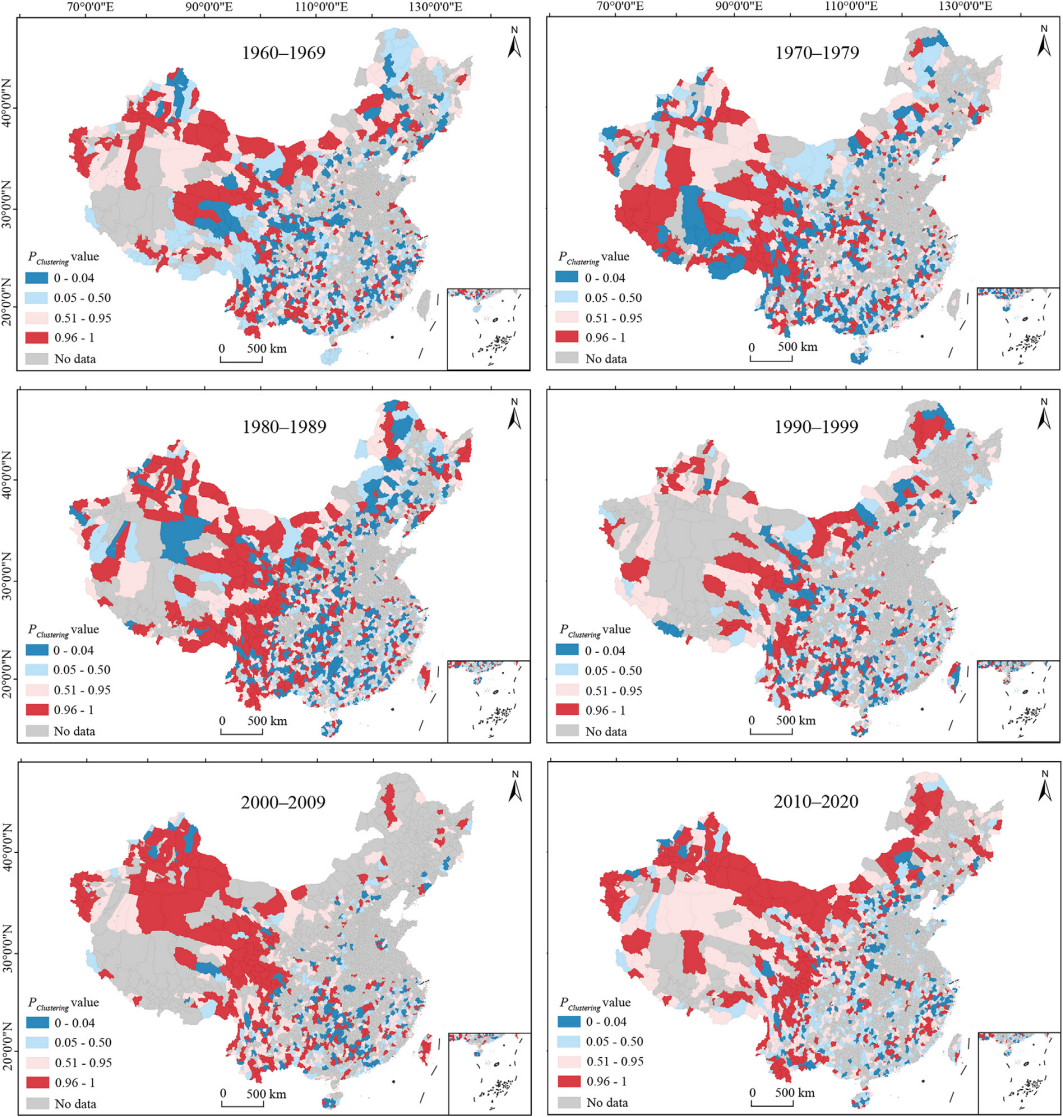
The *P*_*clustering*_ values for the 5 km buffer zones of roads in different counties of China. The *P*_*clustering*_ value indicates the degree of clustering of species occurrence records within specific buffer zones of roads. Each category of *P*_*clustering*_ value corresponds to particular interpretations of the spatial distribution of records in relation to the buffer zones. *P*_*clustering*_ < 0.05: records are significantly situated away from the road buffer zones (left-tailed binomial test, *p* < 0.05); 0.05 ≤ *P*_*clustering*_ < 0.50: fewer records are present within the buffer zones than expected, but this difference is not statistically significant (*p* ≥ 0.05); *P*_*clustering*_ = 0.50: the number of records within the buffer zones is equal to the expected count; 0.50 < *P*_*clustering*_ ≤ 0.95: more records are found within the buffer zones than expected, although this is not statistically significant (*p* ≥ 0.05); *P*_*clustering*_ > 0.95: there is a significant clustering of species occurrence records within the buffer zones (right-tailed binomial test, *p* < 0.05). Maps are Albers projections. Insets in the bottom right of each subplot show the south boundary of China, including all islands in the South China Sea.

At the national level, species occurrence records were disproportionally concentrated within the 5 km and 10 km buffer zones of roads from the 1960s to the 2010s (Fig. 6). The binomial tests indicated a significant clustering of records within the buffer zones, with *P*_*clustering*_ values exceeding 0.99 for all time periods (associated *p* < 0.01). At the county level, counties exhibiting either significantly more or fewer records within the 5 km and 10 km buffer zones than expected (*P*_*clustering*_ > 0.95 or *P*_*clustering*_ < 0.05, *p* < 0.05) tended to be located along mountain ranges, such as the Hengduan, Qilian and Daba Mountains (Figs. 7 and S3).

**Fig. 8:**
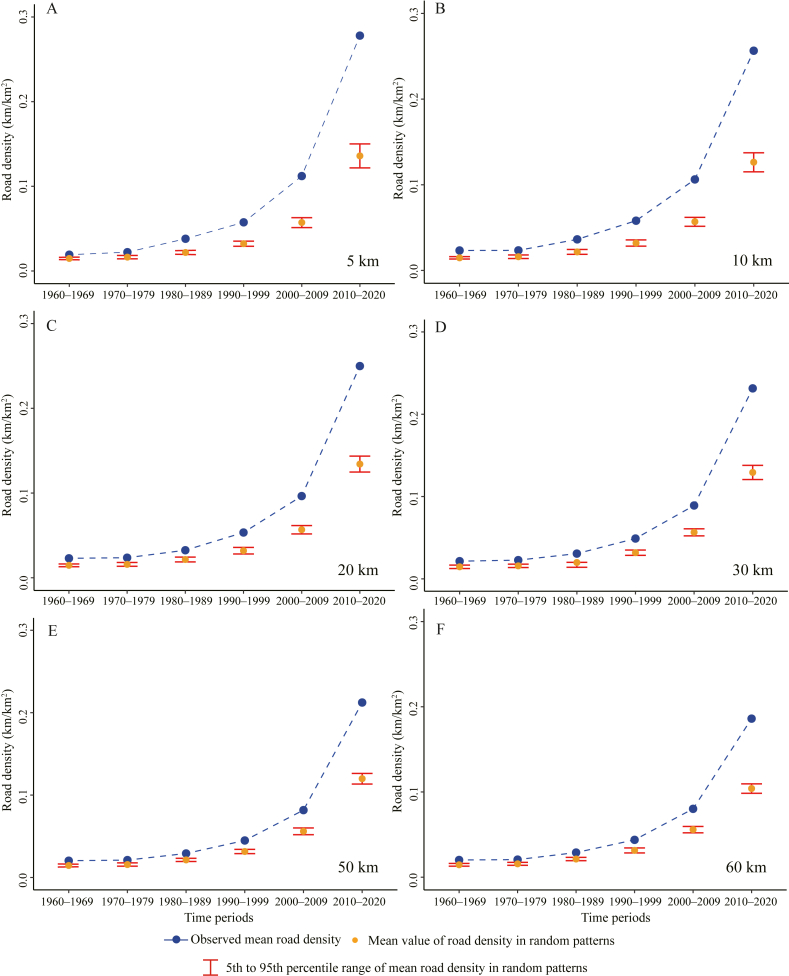
Mean road density within various buffer zones (5–60 km) of species occurrence records in different time periods, compared with road density in random patterns where species occurrence records are randomly distributed over 1000 iterations.

There was an increasing trend in road density surrounding species occurrence records from the 1960s to the 2010s across the 5–60 km buffer zones (Fig. 8). The mean road density within all buffer zones and in all time periods was significantly higher than that of random patterns, indicating that areas with higher road density tended to be more frequently sampled.

### 3.4 Explanatory power of environmental and socio-economic variables on the intensity of the “road-map effect”

The random forest model explained 61.82% of variance in the distance of species occurrence records to the nearest road (Table 1). Elevation range within the 5 km buffer zone of species occurrence records, human population density, annual precipitation, NDVI, and annual mean temperature were selected in the most parsimonious model for the distance to the nearest road. Spatial autocorrelation was eliminated in the model residuals by including three eigenvector-based spatial filters (Fig. S1). The elevation range was found to be the most important variable in the model (Table 1). Elevation range, annual precipitation and NDVI showed positive relationships with the distance to the nearest road, while the other two explanatory variables were negatively correlated.

**Table 1:** Results of the most parsimonious random forest models for three measurements of the “road-map effect”.

Explanatory variables	Pearson's *r*^a^	Relative importance (%)	Overall *R*^2^
Distance to the nearest road^b^			0.62
Elevation range^c^	0.27	24.25	
Human population density	−0.26	21.83	
Annual precipitation	0.03	15.04	
NDVI	0.19	14.90	
Annual mean temperature	−0.19	9.07	
3 spatial filters		14.91	
*P*_*clustering*_ value^d^			0.48
Annual precipitation	−0.14	27.49	
NDVI	−0.19	24.78	
Human population density	0.03	23.99	
Mean elevation^e^	0.12	23.74	
Road density^f^			0.39
Annual precipitation	−0.05	25.79	
Mean elevation^g^	−0.11	25.33	
NDVI	−0.12	25.12	
Human population density	0.18	23.76	

a The Pearson correlation coefficient between the response and explanatory variables.

b The distance of species occurrence records to the nearest road.

c Elevation range within the 5 km buffer zone of species occurrence records.

d *P*_*clustering*_ value indicates the degree of clustering of species occurrence records within the 5 km buffer zone of roads.

e Mean elevation within counties of China.

f Road density within the 5 km buffer zone of species occurrence records.

g Mean elevation within the 5 km buffer zone of species occurrence records. The parameter “ntree” is configured as 2000, 1450, and 1250 for the three random forest models, respectively.

The random forest model accounted for 48.42% of the variance in *P*_*clustering*_ for the 5 km buffer zone (Table 1). Annual precipitation, NDVI, human population density, and mean elevation within counties were included in the most parsimonious model for *P*_*clustering*_. Their relative importance decreased sequentially. Spatial autocorrelation in model residuals was insignificant without including any eigenvector-based spatial filters (Fig. S1). Mean elevation and human population density showed positive relationships with *P*_*clustering*_, while the other selected explanatory variables exhibited negative relationships (Table 1). Similar results of random forest modeling were obtained for *P*_*clustering*_ within the 10 km buffer zone (Table S1).

The random forest model explained 39.35% of the variance in road density within the 5 km buffer zone of species occurrence records (Table 1). The most parsimonious model for road density included annual precipitation, mean elevation within the 5 km buffer zone of species occurrence records, NDVI, and human population density. Their importance in the model decreased successively. With the exception of human population density, all other selected variables exhibited negative relationships with road density. Similar results were obtained for the road density within the 10 km buffer zone of species occurrence records (Table S1).

## 4 Discussion

### 4.1 Road network expansion in China

The road network of China expanded rapidly from 1960 to 2020, with particularly accelerated growth after the year 2005 (Fig. 1A). This is closely linked to the rapid economic development and the acceleration of urbanization in China (Zhou et al., 2022). The road network expansion in China can be divided into two stages. The first stage is from 1960 to 2004, during which the road mileage showed a steady growth (Fig. 1A). In this period, the total road mileage increased from 1,351,691 km in 1999 to 1,679,848 km in 2000. This considerable growth is due to the inclusion of low-class roads (low-speed minor roads) in the national road census conducted in 2000, which may have been overlooked in earlier road mileage calculations (Ministry of Transport of the People's Republic of China, 2005). The second stage began in 2005, during which the growth rate of road mileage accelerated, which can be attributed to the rapid development of highway network and the implementation of the Village to Village Roads Project (Wong et al., 2017). Notably, between 2004 and 2005, there was a 1,474,500 km increase in road mileage. This growth was a result of a modification in statistical criteria from 2005 onwards, now encompassing village roads in the calculation of total road mileage.

Road density has been consistently higher in the eastern regions compared to the western regions since the 1960s (Fig. S2), which is strongly determined by the human population density, i.e., areas with greater population density generally have higher road density (Hu et al., 2018). This spatial pattern has become increasingly prominent in recent decades, with a notably greater increase in road mileage in the eastern and southeastern regions (Fig. 1B), attributed to the rapid economic development in these regions. The rapid expansion of road networks could induce stronger human-caused disturbances to the natural ecosystems, particularly in the eastern and southern regions, which are renowned for their high biodiversity (Huang et al., 2011). Addressing the impact of road expansion on natural ecosystems and biodiversity remains a substantial challenge in the future (Kleinschroth et al., 2019).

### 4.2 Variation in floristic collecting efforts over the past decades

The number of species occurrence records from the 1960s to the 1980s is higher than that from the 1990s and 2010s, and peaked in the 1980s (Fig. 2). This temporal trend is consistent with the overall trend of the intensity of floristic collections in China, which is largely influenced by national policies (Yang et al., 2013). One peak period for floristic collections in China was from 1958 to 1962, during which extensive plant resource surveys was conducted nationwide with the aim of understanding the distribution of all plant species, especially economic plants (e.g., edible or medicinal plants; Yang et al., 2021). Relatively fewer plant specimens were collected in the 1960s due to the influence of the Cultural Revolution (Chen, 1994). In 1973, the central government initiated the first comprehensive scientific investigation on the Qinghai-Tibet Plateau (1973–1980) with the aim of gathering fundamental information on natural resources, including plant resources. The comprehensive scientific survey of the Hengduan Mountains was conducted from 1980 to 1985, during which a large number of plant specimens were collected.

However, the number of plant specimens has remained relatively low since the 1990s (Liu et al., 2022b), largely due to decreased research funding for traditional taxonomy, as resources have shifted towards disciplines such as cytology and molecular biology. Due to the notable geographical and taxonomic biases in current collecting efforts and the lack of complete species checklists in the majority of counties in China (Yang et al., 2021), it is necessary to continue field surveys and floristic collections in China, especially focusing on regions and species that have been underrepresented in current biological collections.

Floristic collections showed similar geographical distributions in all decades (Fig. 3), with a large proportion of these collections concentrating in the prominent mountain ranges (e.g., Hengduan, Daba and Nanling Mountains). These mountains are renowned for their rich biodiversity (Qian and Kissling, 2010; Wang et al., 2012), making them a preferred destination for collectors seeking to encounter a diverse array of species (Loiselle et al., 2008). In contrast, lowland areas (e.g., North China Plain and Sichuan Basin) are underrepresented in the floristic collections. These areas have experienced stronger human disturbances compared to mountainous areas (Zhang et al., 2022). As a result, biodiversity in lowlands is subjected to greater threats, including biological invasion, the decline of native species, and the homogenization of biological communities (Šipek and Šajna, 2024). It is therefore crucial to enhance biological surveys in these areas to improve our understanding of the impact of human activities on biodiversity. Our findings are different from the global patterns that suggest the availability of species occurrence records primarily determined by proximity to researchers and local research funding (Meyer et al., 2015). This implies that the factors influencing spatial patterns in biological survey efforts might be specific to particular regions or countries.

### 4.3 “Road-map effect” on floristic collections of China

The mean distance of species occurrence records to the nearest road was significantly shorter than that in random patterns across all time periods (Fig. 4), suggesting that areas closer to roads are more likely to be sampled (Crisp et al., 2001; Petersen et al., 2021). In addition, the distance to the nearest road declined from the 1960s to the 2010s, suggesting that plant specimen collections have been increasingly closer to roads. This is partly due to the gradual densification of road networks, increasing the likelihood of species occurrence records being closer to roads. Furthermore, our results showed that the reduction in the observed mean distance from the 1960s to the 2010s was disproportionally faster than the reduction in the mean distance derived from random patterns (Fig. 4, Fig. 5A). This suggests that collectors have increasingly stayed closer to roads, even after accounting for the influence of road network expansion.

Previous studies have explored the spatial relationships between species occurrence records and road networks, often neglecting the temporal dynamics of these relationships (Freitag et al., 1998; Parnell et al., 2003). In this study, we correlated the records collected in different decades with the corresponding road networks of those decades. This approach facilitated a more accurate assessment of the “road-map effect” and revealed a temporal trend that biological sampling has increasingly relied on road networks, a pattern seldom reported in earlier studies.

Elevation range, human population density, annual precipitation, NDVI, and annual mean temperature are all important in modelling the distance to the nearest road (Table 1). Human population density and annual mean temperature exhibited negative correlations, while the remaining variables demonstrated positive correlations with the distance to the nearest road. This suggests that in mountainous areas well covered with vegetation, collectors tend to stray further away from roads for collecting specimens. Compared to lowland areas with similar climatic conditions, mountainous areas provide diverse habitats for biological organisms and therefore foster greater species diversity (Wang et al., 2012; Badgley et al., 2017). Additionally, the low human population density implies minimal human disturbances. In such areas (e.g., the mountainous areas in southwestern China), collectors are likely to discover a wider range of species (including unique and rare species), and thus motivated to explore the areas farther away from roads (Loiselle et al., 2008).

Mean elevation was positively correlated with the *P*_*clustering*_ values, while annual precipitation and NDVI were negatively correlated with them (Tables 1 and S1). Low precipitation and NDVI indicate low species diversity and sparse vegetation cover (Liu et al., 2022a; Coelho et al., 2023). This suggests that in high elevation areas with low species diversity and vegetation cover, collectors often concentrate their sampling efforts along roadside areas (Crisp et al., 2001; Daru et al., 2018). This is evidenced by the spatial distribution of counties with *P*_*clustering*_ values larger than 0.95 in the relatively dry plateau regions (Figs. 7 and S3). In contrast, counties with *P*_*clustering*_ values less than 0.05 were primarily located along mountain ranges, such as the Daba and Nanling Mountains. These mountainous regions, characterized by relatively high precipitation and NDVI, generally display greater species diversity, which makes them more attractive to collectors, resulting in more surveys being conducted away from roads.

The mean road density within various buffer zones (5–60 km) of species occurrence records was significantly higher than that of random patterns in all time periods (Fig. 8), which is consistent with previous findings that areas with higher accessibility tend to be more frequently sampled (Oliveira et al., 2016). Human population density was positively correlated with road density, while mean elevation is negatively correlated with it. This is likely due to the denser road network in the highly populated and lowland regions of China. Annual precipitation and NDVI were negatively correlated with the road density surrounding species occurrence records, suggesting that collectors tend to sample in easily accessible locations within regions of lower vegetation coverage. Fewer species are expected to be found in these regions, hindering enthusiasm to venture into less accessible locations.

Our findings reveal a significant “road-map effect” in the floristic collections of China, and the intensity of this effect increased from the 1960s to the 2010s. Elevation range, mean elevation, annual precipitation, NDVI, and human population density were identified as crucial factors for predicting the intensity of the “road-map effect” across various regions in China. Adequate rainfall, complex topography, and dense vegetation facilitate greater species diversity (Irl et al., 2015), motivating collectors to explore remote, roadless locations for sampling in such areas (Loiselle et al., 2008). In contrast, collectors tend to prioritize sampling locations near roads in sparsely vegetated mountainous areas, as the rugged terrain makes accessing remote locations difficult. Human population density signifies the degree of disruption caused by human activities (Ai et al., 2024). The positive effect of human population density on the intensity of “road-map effect” suggests that collectors may prefer to sample the areas with more pristine vegetation (Yang et al., 2014).

## 5 Conclusion

Species occurrence records collected in different time periods showed similar geographical patterns, with a large proportion of these records located along mountain ranges, while the lowland areas are much underrepresented. A notable intensification of “road-map effect” was identified from the 1960s to the 2010s, suggesting an increased reliance of biological surveys on road networks even after controlling for the influence of road network expansion, which has rarely been reported in published studies. Specifically, species occurrence records of vascular plants have been increasingly concentrated nearer to roads. The road density surrounding the records has progressively increased, indicating that accessibility plays a growing role in determining the likelihood of an area being sampled. Environmental and socio-economic variables affecting regional species diversity, vegetation cover, and the pristine state of vegetation, all of which influence willingness to invest additional efforts in surveying locations situated far from roads, played important roles in predicting the intensity of the “road-map effect”. These results indicate that future floristic surveys in China should focus on lowland areas and remote areas characterized by low road density and considerable distances from roads. Our study also suggests that given the prevalence of the “road-map effect” in species occurrence data, it is important to account for it when using such data for both theoretical and practical purposes.

## CRediT authorship contribution statement

**Jingyang He:** Writing – original draft, Visualization, Validation, Software, Methodology, Formal analysis. **Wenjing Yang:** Writing – review & editing, Supervision, Resources, Methodology, Investigation, Conceptualization. **Qinghui You:** Writing – review & editing, Resources, Methodology, Investigation, Funding acquisition, Data curation. **Qiwu Hu:** Writing – review & editing, Validation, Methodology, Data curation. **Mingyang Cong:** Writing – review & editing, Visualization, Methodology, Data curation. **Chao Tian:** Writing – review & editing, Validation, Software. **Keping Ma:** Writing – review & editing, Resources, Project administration, Investigation, Data curation.

## Availability of data and materials

The datasets that support the findings of this study are available on request from the corresponding author, upon reasonable request.

## Declaration of competing interest

These authors have no conflict in interest.
