# Supplementary material for: Impacts of road networks on the geography of floristic collections in China
Source: Plant Divers. 2025 Feb 21;47(3):403–14. doi: 10.1016/j.pld.2025.02.001 (PMC12146856; doi:10.1016/j.pld.2025.02.001)
Supplement: Multimedia component 1 [file mmc1.docx]

**Appendix A. Supplementary data for**

**Title: Impacts of road networks on the geography of floristic collections in China**

**Authors: Jingyang He, Wenjing Yang, Qinghui You, Qiwu Hu, Mingyang Cong, Chao Tian, Keping Ma**

^The supplementary files including three figures, one table and one document for r code.^


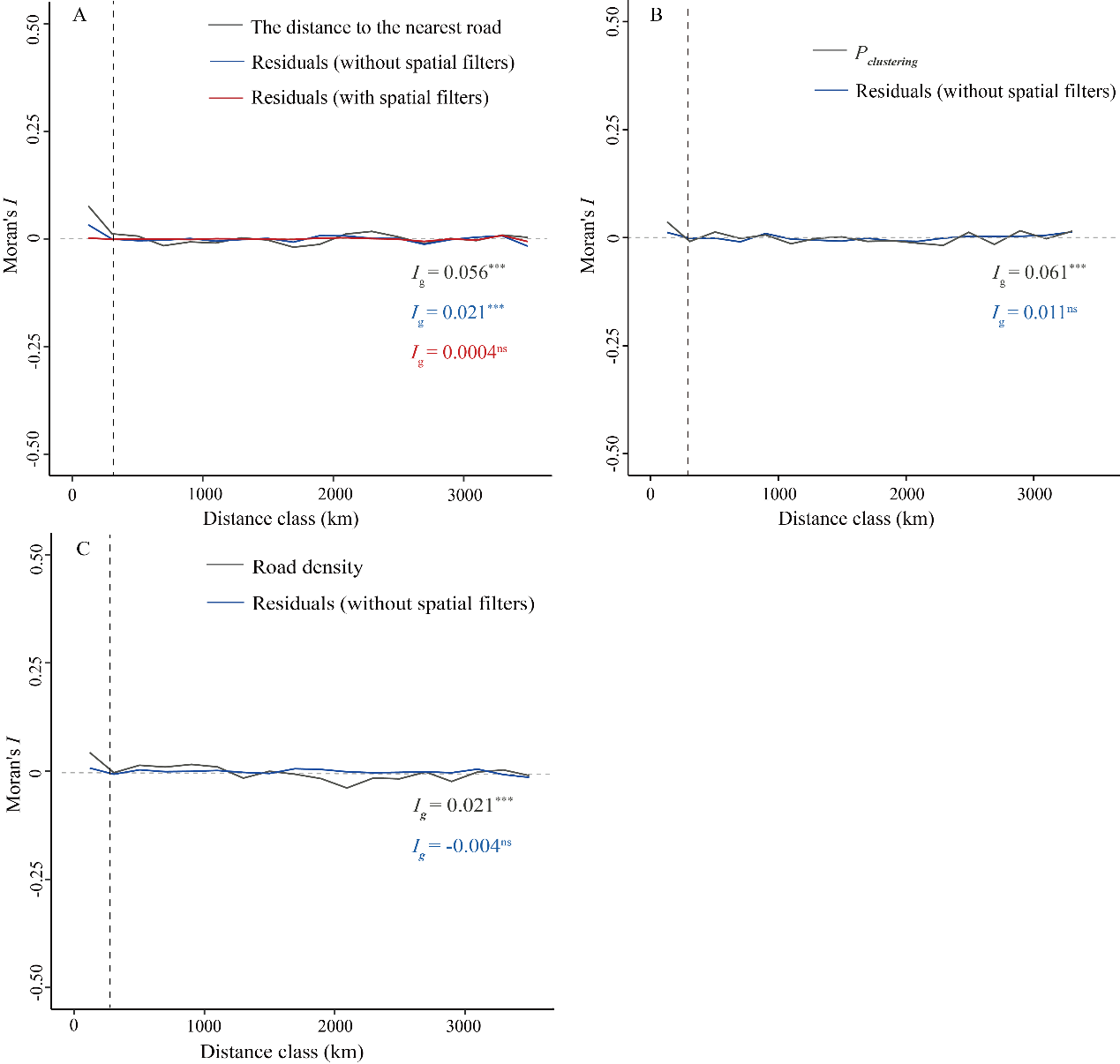


**Fig. S1.** Moran's *I* correlograms for the distance of species occurrence records to the nearest road (A, gray), *P_clustering_* value (B, gray), road density within the 5 km buffer zone of species occurrence records (C, gray), and residuals from the random forest models without (blue) and with spatial filters (red). The *P_clustering_* value indicates the degree of clustering of species occurrence records within the 5 km buffer zone of roads. The vertical dashed lines represent a distance of 300 km. Global Moran's *I* value (*I_g_*, calculated with a neighbor distance of 300 km) significance: *** < 0.001; ^ns^ not significant.

**
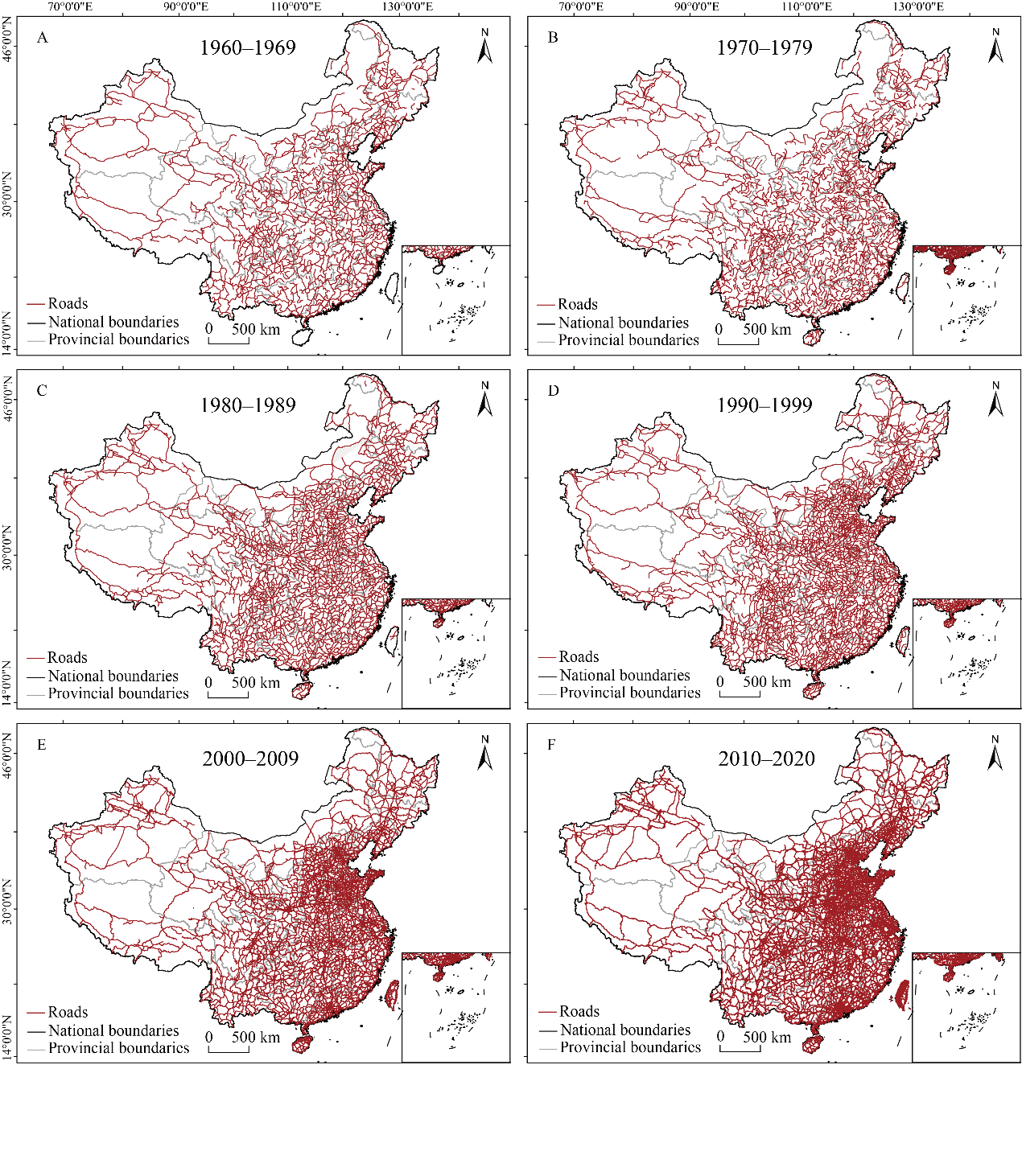
**

**Fig. S2.** Road networks in China across different time periods. Maps are Albers projections. Insets in the bottom right of figures show the south boundary of China, including all islands in the South China Sea.

**
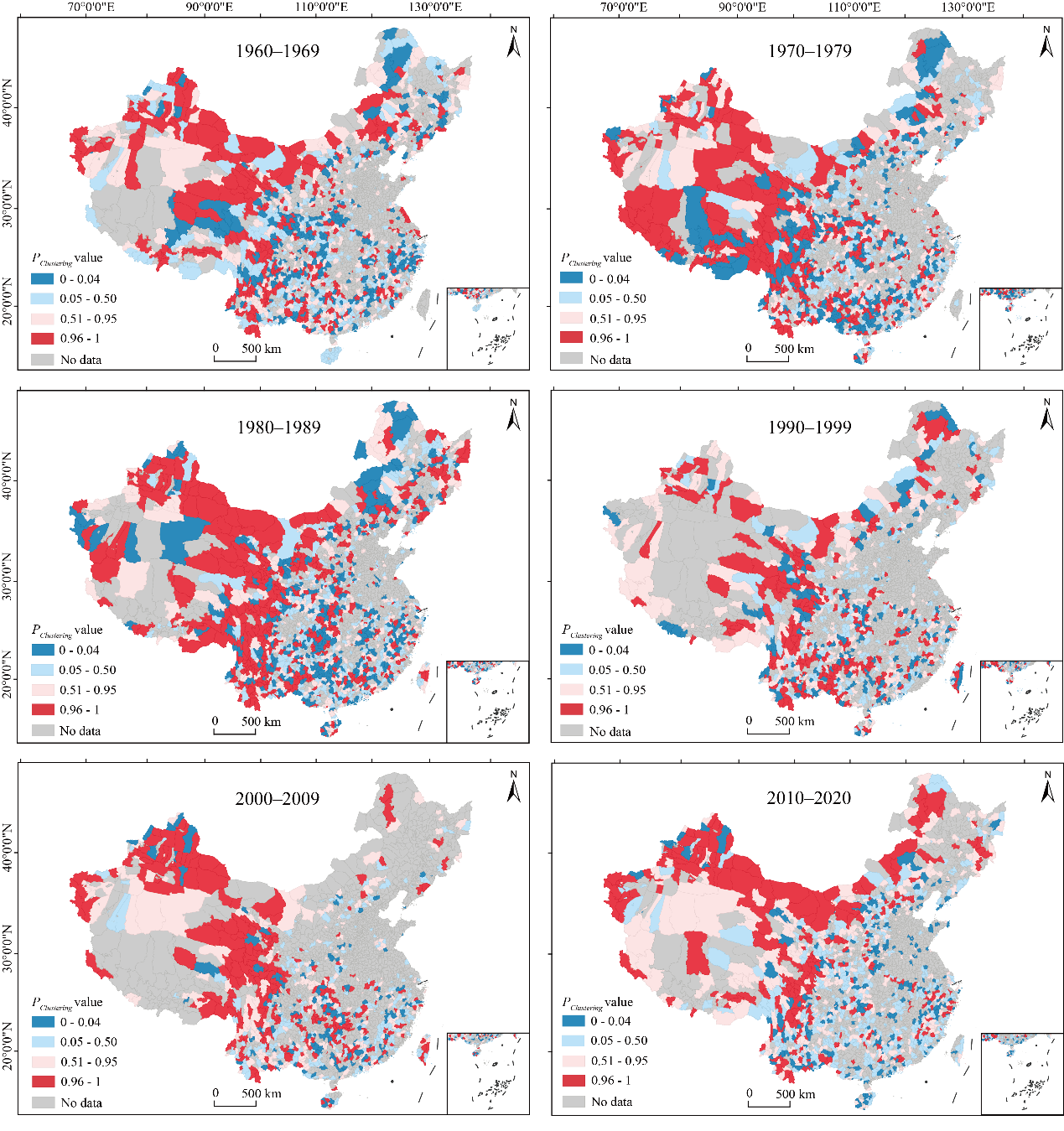
**

**Fig. S3** The *P_clustering_* values for the 10 km buffer zones of roads in different counties of China. The *P_clustering_* value indicates the degree of clustering of species occurrence records within specific buffer zones of roads. Each category of *P_clustering_* value corresponds to particular interpretations of the spatial distribution of records in relation to the buffer zones. *P_clustering_* < 0.05: records are significantly situated away from the road buffer zones (left-tailed binomial test, *p* < 0.05); 0.05 ≤ *P_clustering_* < 0.50: fewer records are present within the buffer zones than expected, but this difference is not statistically significant (*p* ≥ 0.05); *P_clustering_* = 0.50: the number of records within the buffer zones is equal to the expected count; 0.50 < *P_clustering_* ≤ 0.95: more records are found within the buffer zones than expected, although this is not statistically significant (*p* ≥ 0.05); *P_clustering_* > 0.95: there is a significant clustering of species occurrence records within the buffer zones (right-tailed binomial test, *p* < 0.05). Maps are Albers projections. Insets in the bottom right of each subplot show the south boundary of China, including all islands in the South China Sea.

**Table S1.** Results of the most parsimonious random forest models for *P_clustering_* values and road density within the 10 km buffer zone of roads. The *P_clustering_* value indicates the degree of clustering of species occurrence records within the 10 km buffer zone of roads.

| Explanatory variables | Pearson's *r*^a^ | Relative importance (%) | Overall *R*^2^ |
| --- | --- | --- | --- |
| *P_clustering_* value |  |  | 0.54 |
| Annual precipitation | -0.19 | 26.43 |  |
| NDVI | -0.27 | 25.71 |  |
| Human population density | 0.04 | 25.28 |  |
| Mean elevation^b^ | 0.16 | 22.58 |  |
| Road density |  |  | 0.46 |
| Human population density | 0.12 | 16.82 |  |
| Mean elevation^c^ | -0.08 | 15.49 |  |
| NDVI | -0.11 | 14.01 |  |
| Annual precipitation | -0.05 | 12.54 |  |
| 3 spatial filters | - | 27.78 |  |

^a^ the Pearson correlation coefficient between the response and explanatory variables; ^b^ mean elevation within counties of China; ^c^ mean elevation within the 10 km buffer zone of species occurrence records. The parameter “ntree” is configured as 1750 and 1250 for the two random forest models, respectively.

**The r code for three methods of quantifying the intensity of road-map effect**

######### First Method for quantifying the road-map effects ################

##### Quantifying road-map effects using shortest distance method ##############

##### Example frequency distribution histogram and gamma function fitting ######

# Load the required library

library(MASS)

distance <- read.csv("D:/Example Code/distance.csv")

###########################example data####################

distance <- c(

36.66613839, 17.33840944, 11.60007969, 15.19626536, 21.9865877, 3.490661621,

10.69000415, 7.397338293, 6.970680584, 7.942350586, 1.882449572, 25.2280705,

15.09250508, 22.50450137, 29.14075882, 1.595512258, 14.89263384, 1.03274147,

35.69822021, 2.383326101, 28.33433908, 37.82738754, 11.84340188, 5.524310792,

1.117784057, 30.78787816, 33.71018042, 8.282309349, 11.89563589, 31.92640369,

28.40223077, 2.078727342, 6.353241877, 17.41596731, 31.64867759, 21.12459546,

20.35344672, 21.54773882, 1.556747192, 7.296603817, 27.27661492, 9.130647644,

14.50282223, 10.13830211, 18.88658783, 36.81573177, 18.64111859, 3.891311753,

5.951198173, 18.36043939, 34.36939592, 11.73060169, 2.57115658, 8.454699414,

3.22668418, 16.29720825, 6.943374888, 21.44271269, 33.65786074, 22.00444513,

1.827167509, 9.823295922, 17.70782125, 7.763421784, 13.10890761, 29.98722194,

28.85115405, 8.96733397, 2.136229207, 13.1408765, 5.468727351, 10.86432569,

26.55188422, 2.068935378, 1.967835924, 4.056139969, 22.57545224, 31.91980014,

4.395970984, 25.31966151, 12.22187431, 35.67158056, 11.76061476, 14.3432084,

17.02829989, 0.965610275, 7.848915043, 36.02152399, 14.17302703, 5.89476299,

7.968003193, 3.056152188, 26.31471416, 3.196531514, 33.28837792, 15.21077116,

6.65531286, 19.91841894, 9.646816076, 0.742757357, 59.99883107, 56.23559511,

64.57668989, 64.50939965, 49.46071534, 56.64191895, 41.89859074, 47.25220472,

60.04426797, 60.1965187, 42.43727157, 60.82555846, 63.98354193, 41.53632001,

40.15545631, 65.42142521, 41.08742011, 76.34975634, 50.95928954, 60.6283073,

55.32907917, 63.23681108, 41.27837587, 41.46297218, 91.58711893, 84.43772399,

89.5119585, 88.564599, 82.16049669, 82.50509947, 110.2634097, 107.1287744,

79.5895219, 105.305568, 95.23335682, 101.1043316, 115.9070261, 139.8968585,

143.6285449, 131.0923894, 136.498393, 147.0530778, 155.1318054, 171.3190183,

163.5963314, 208.3367352, 194.1115808, 257.5296311, 280.6451182, 6.016364366

)

# Plot the histogram

hist(distance, nclass = 30, xlim = c(0, 200))

# Gamma fitting parameter estimation

x <- distance

fit <- fitdistr(x, "gamma", lower = 0.001)

# Extract Gamma distribution parameters

shape <- fit$estimate["shape"] # Shape parameter

rate <- fit$estimate["rate"] # Rate parameter

# Here y1 is undefined; we assume it is for performing a KS test to compare with the Gamma distribution density

y1 <- dgamma(x, shape = shape, rate = rate)

ks_result <- ks.test(x, "pgamma", shape = shape, rate = rate)

print(ks_result)

# Calculate Gamma distribution density

y <- dgamma(x, shape = shape, rate = rate)

x1 <- x[order(x)]

y1 <- y[order(x)]

# Plot the histogram with the fitted curve

histogram <- hist(x, nclass = 30, freq = FALSE,

xlab = "Distance (km)",

main = "Shortest Distance Frequency Distribution",

ylim = c(0, 0.03),

xlim = c(0, 200),

right = FALSE)

lines(x1, y1, col = "red", lwd = 2)

a <- mean(x)

abline(v = a, col = "green", lty = 2, lwd = 2)

######################### Second method #############################

############## Bias value calculation to quantify road network effects ##########

######## Using a 5 km buffer around roads as an example #####################

library(sf)

library(sp)

library(dplyr)

########### Step 1: Calculate the number of observation points in each county in ArcGIS, use this as a basis, generate random points corresponding to the number of observation points in each county, and export them as a shapefile (shp) file. #######

Observation_Count<-read_excel("D:/Example_Code/sum_actualPoint_xian.xlsx")

################# Example data: observation count per county ###############

FID NAME FREQUENCY

1 Dengfeng Shi 1

2 Dengzhou Shi 1

3 Erqi Qu 2

4 Fengqiu Xian 122

5 Gongyi Shi 3

6 Gushi Xian 2

7 Gulou Qu 2

8 Guancheng Huizu Qu 4

9 Guancheng Huizu Qu 1

10 Hongqi Qu 1

11 Huangchuan Xian 11

12 Huixian Shi 5

13 Huiji Qu 1

14 Jinshui Qu 2

15 Jiyuan Shi 51

16 Lingbao Shi 1

17 Longting Qu 1

18 Lushi Xian 12

19 Luanchuan Xian 7

20 Luohe Xian 1

21 Luolong Qu 2

22 Miyang Xian 1

23 Muyuan Qu 4

24 Nanzhao Xian 3

25 Neixiang Xian 3

26 Pingqiao Qu 3

27 Qi Xian 1

28 Shangcheng Xian 49

29 Shihe Qu 89

30 Shunhe Huizu Qu 1

31 Song Xian 12

32 Shangjie Qu 12

33 Tongbai Xian 87

34 Tongxu Xian 1

35 Wuzhi Xian 1

36 Xiangfu Qu 80

37 Xingyang Shi 3

38 Xinmi Shi 4

39 Xinxian 53

40 Xinyang Xian 30

41 Xiuwu Xian 1

42 Xinzheng Shi 1

43 Yicheng Qu 1

44 Yicheng Qu 1

45 Yuzhou Shi 1

46 Yuwangtai Qu 1

47 Zhongmu Xian 4

48 Zhongyuan Qu 1

1. Zhenping Xian 80
2. Zhongyuan Qu 1

county_boundaries <- readOGR(dsn = "D:/Example_Code/County_Boundaries.shp", layer = "County_Boundaries")

county_data <- Observation_Count

random_points <- data.frame(lon = numeric(), lat = numeric())

for (i in 1:nrow(county_data)) {

county_name <- county_data[i, "NAME"]

frequency <- county_data[i, "FREQUENCY"]

# Check if the frequency is a positive integer

if (is.numeric(frequency) && frequency > 0) {

county_boundary <- county_boundaries %>%

filter(NAME == county_name)

# Check if the county boundary exists

if (!is.null(county_boundary)) {

county_random_points <- st_sample(county_boundary, size = as.integer(frequency), type = "random")

# Extract the coordinates of the points

county_random_points_coords <- st_coordinates(county_random_points)

# Convert the coordinates to a data frame

county_random_points_df <- as.data.frame(county_random_points_coords)

# Append the current county's random points to the total random points data frame

random_points <- rbind(random_points, county_random_points_df)

print(paste("Processing county:", county_name))

print(paste("Frequency:", frequency))

} else {

message(paste("Warning: No boundary found for county:", county_name))

}

} else {

message(paste("Warning: Invalid frequency value for county:", county_name, "- skipping sampling"))

}

}

# Display and export the random points shapefile

random_points_sf <- st_as_sf(random_points, coords = c("X", "Y"), crs = st_crs(county_boundaries))

plot(random_points_sf)

st_write(random_points_sf,"D:/Example_Code/County_Random_Points_Example.shp")

############ Calculate and summarize the number of actual observation points and random points within the road buffer for each county in ArcGIS #################

################# Join the total sample points per county, the number of actual points within the buffer, and the number of random points into a table #############

# Example data of the join result

NAME sum_point sample_point actual_point

1 Dengfeng Shi 1 1 0

2 Dengzhou Shi 1 0 0

3 Erqi Qu 2 2 0

4 Fengqiu Xian 122 60 20

5 Gongyi Shi 3 2 1

6 Gushi Xian 2 1 0

7 Gulou Qu 2 2 0

8 Guancheng Huizu Qu 4 3 0

9 Guancheng Huizu Qu 1 0 0

10 Hongqi Qu 1 0 0

11 Huangchuan Xian 11 3 1

12 Huixian Shi 5 3 2

13 Huiji Qu 1 0 0

14 Jinshui Qu 2 2 0

15 Jiyuan Shi 51 46 23

16 Lingbao Shi 1 0 0

17 Longting Qu 1 1 0

18 Lushi Xian 12 5 1

19 Luanchuan Xian 7 6 1

20 Luohe Xian 1 1 0

21 Luolong Qu 2 2 0

22 Miyang Xian 1 0 0

23 Muyuan Qu 4 0 0

24 Nanzhao Xian 3 1 0

25 Neixiang Xian 3 2 1

26 Pingqiao Qu 3 2 1

27 Qi Xian 1 1 0

28 Shangcheng Xian 49 36 12

29 Shihe Qu 89 85 23

30 Shunhe Huizu Qu 1 1 0

31 Song Xian 12 3 0

32 Shangjie Qu 12 5 1

33 Tongbai Xian 87 65 11

34 Tongxu Xian 1 0 0

35 Wuzhi Xian 1 1 0

36 Xiangfu Qu 80 23 34

37 Xingyang Shi 3 2 1

38 Xinmi Shi 4 3 0

39 Xinxian 53 11 2

40 Xinyang Xian 30 1 9

41 Xiuwu Xian 1 0 1

42 Xinzheng Shi 1 0 0

43 Yicheng Qu 1 0 1

44 Yicheng Qu 1 0 1

45 Yuzhou Shi 1 0 0

46 Yuwangtai Qu 1 0 0

47 Zhongmu Xian 4 0 0

48 Zhongyuan Qu 1 1 0

49 Zhenping Xian 80 23 34

50 Zhongyuan Qu 1 1 0

############# Significance calculation code #############

library(dplyr)

library(broom)

# p5 is obtained based on the ratio of road area within the county to the total county area, and similarly associated

#example_data

NAME sum_point sample_point actual_point xian_area buf_area p5

1 Dengfeng Shi 1 1 0 197805417.5 10403837.86 0.052596324

2 Dengzhou Shi 1 0 0 155967909.8 37246024.49 0.238805691

3 Erqi Qu 2 2 0 993710663.8 239980847.3 0.24149972

4 Fengqiu Xian 122 60 20 199334934.8 99459112.88 0.498954752

5 Gongyi Shi 3 2 1 768618517.3 0 0

6 Gushi Xian 2 1 0 242006448.3 52936867.71 0.218741559

7 Gulou Qu 2 2 0 62866802.32 62866802.32 1

8 Guancheng Huizu Qu 4 3 0 56817023.82 0 0

9 Guancheng Huizu Qu 1 0 0 886034465.1 475335248.5 0.536474897

10 Hongqi Qu 1 0 0 222146263.9 2084125.102 0.00938177

11 Huangchuan Xian 11 3 1 1420586217 0 0

12 Huixian Shi 5 3 2 1039924940 159110195.9 0.153001616

13 Huiji Qu 1 0 0 60214784.44 19884876.38 0.33023246

14 Jinshui Qu 2 2 0 993710663.8 239980847.3 0.24149972

15 Jiyuan Shi 51 46 23 947981569.2 0 0

16 Lingbao Shi 1 0 0 886034465.1 475335248.5 0.536474897

17 Longting Qu 1 1 0 72501619.11 9549126.283 0.131709145

18 Lushi Xian 12 5 1 1218483144 201868010 0.165671566

19 Luanchuan Xian 7 6 1 366465799.2 155689627.4 0.424840811

20 Luohe Xian 1 1 0 72501619.11 9549126.283 0.131709145

21 Luolong Qu 2 2 0 62866802.32 62866802.32 1

22 Miyang Xian 1 0 0 60214784.44 19884876.38 0.33023246

23 Muyuan Qu 4 0 0 1247123311 157611724.2 0.126380225

24 Nanzhao Xian 3 1 0 1258767135 424729027.9 0.33741668

25 Neixiang Xian 3 2 1 768618517.3 0 0

26 Pingqiao Qu 3 2 1 1301070049 404549094.5 0.310935675

27 Qi Xian 1 1 0 2304992304 653262139.1 0.283411853

28 Shangcheng Xian 49 36 12 1104528432 260619612.4 0.235955549

29 Shihe Qu 89 85 23 53302024.97 52615351.3 0.987117306

30 Shunhe Huizu Qu 1 1 0 53460630.81 43766973.78 0.818676718

31 Song Xian 12 3 0 25618653.36 17688432.61 0.690451304

32 Shangjie Qu 12 5 1 1218483144 201868010 0.165671566

33 Tongbai Xian 87 65 11 79173247.37 73533076.42 0.928761657

34 Tongxu Xian 1 0 0 60214784.44 19884876.38 0.33023246

35 Wuzhi Xian 1 1 0 80631183.29 19909381.89 0.246919133

36 Xiangfu Qu 80 23 34 1616503896 497553016.5 0.307795742

37 Xingyang Shi 3 2 1 1301070049 404549094.5 0.310935675

38 Xinmi Shi 4 3 0 56817023.82 0 0

39 Xinxian 53 11 2 729661496.4 215599168.5 0.295478341

40 Xinyang Xian 30 1 9 1165107620 20441805.15 0.017544993

41 Xiuwu Xian 1 0 1 2475512543 320761546.7 0.129573792

42 Xinzheng Shi 1 0 0 222146263.9 2084125.102 0.00938177

43 Yicheng Qu 1 0 1 3007623088 548372219.3 0.18232744

44 Yicheng Qu 1 0 1 3007623088 548372219.3 0.18232744

45 Yuzhou Shi 1 0 0 1327622331 153992533 0.115991219

46 Yuwangtai Qu 1 0 0 1327622331 153992533 0.115991219

47 Zhongmu Xian 4 0 0 1247123311 157611724.2 0.126380225

48 Zhongyuan Qu 1 1 0 2304992304 653262139.1 0.283411853

49 Zhenping Xian 80 23 34 1616503896 497553016.5 0.307795742

50 Zhongyuan Qu 1 1 0 2304992304 653262139.1 0.283411853

colnames(example_data) <- c("NAME", "N", "k", "p")

counties_data1 <- example_data

# Add a mean column for subsequent judgment

counties_data <- counties_data1 %>%

mutate(E = p * N, # Calculate the E column

proximity_category = NA,

p_value = NA) # Initialize the class column

calculate_p_value <- function(k, N, p) {

if (is.na(k) || is.na(N) || is.na(p) || N < 0 || k < 0 || p < 0 || p > 1) {

return(NA)

}

if (k > N) {

stop("Error: 'k' cannot be greater than 'N'.")

}

if (k >= N * p) {

test_result <- binom.test(k, N, p, alternative = "greater")

} else {

test_result <- binom.test(k, N, p, alternative = "less")

}

return(test_result$p.value)

}

head(counties_data)

# Perform data classification for each county

for (i in 1:nrow(counties_data)) {

k <- counties_data$k[i]

N <- counties_data$N[i]

p <- counties_data$p[i]

E <- counties_data$E[i]

# Check if N and p are zero, and if E is zero

if (N == 0 || p == 0 || E == 0) {

counties_data$p_value[i] <- NA

counties_data$proximity_category[i] <- NA

next

}

# Calculate the p-value

p_val <- calculate_p_value(k, N, p)

counties_data$p_value[i] <- p_val

# Classify proximity_category

if (!is.na(p_val)) {

if (k > E && p_val < 0.05) {

counties_data$proximity_category[i] <- "Significantly Close"

} else if (k < E && p_val < 0.05) {

counties_data$proximity_category[i] <- "Significantly Far"

} else if (k > E && p_val > 0.05) {

counties_data$proximity_category[i] <- "Not Significantly Close"

} else if (k < E && p_val > 0.05) {

counties_data$proximity_category[i] <- "Not Significantly Far"

} else if (k == E) {

counties_data$proximity_category[i] <- "Not Significantly Close"

} else {

counties_data$proximity_category[i] <- NA

}

} else {

counties_data$proximity_category[i] <- NA

}

}

write.csv(counties_data, "D:/Example_Code/Bias_result.csv")

# Subsequent steps involve classification in Excel and visualization in ArcGIS #####

###########################Third method ##############################

############# Quantifying road network effects using road density #############

#First, generate 1000 random point files, with the number of random points matching the specimen data volume for each corresponding decade.

# Calculate the average road density within sample buffer zones in ArcGIS and compute quantiles. # Use R for plotting.

library(sf) # Load the sf package

library(sp) # Load the sp package (if not already loaded)

library(rgdal)

# Read the shapefile (assuming it is a SpatialPolygonsDataFrame)

border <- readOGR(dsn = "D:/Example_Code/Provincial_Boundary_example.shp")

# Convert SpatialPolygonsDataFrame to an sf object

border_sf <- st_as_sf(border)

# Set random seed

set.seed(123)

# Parameter settings

n_files <- 10 # Number of files

n_points <- 300 # Number of random points per file

# Ensure the boundary data has a CRS

if (is.na(st_crs(border_sf))) {

stop("Boundary data lacks CRS. Please check the input file or manually set the CRS.")

}

# Loop to generate random points and save

for (i in 1:n_files) {

# Generate random points within the boundary

random_points <- st_sample(border_sf, size = n_points, type = "random")

# Convert sfc to an sf object and inherit the CRS

random_points_sf <- st_as_sf(random_points)

st_crs(random_points_sf) <- st_crs(border_sf) # Inherit the CRS from the boundary data

# Save as a shapefile

filename <- paste0("D:/Example_Code/random_points_", i, "_60s.shp")

st_write(random_points_sf, filename)

# Print progress

cat("File generated:", filename, "\n")

}

# After generating the random point files, calculate road density within buffers in ArcGIS for the 1000 random point files.

# Compute the mean, median, quantiles, etc.

# Plotting

library(ggplot2)

data_60km <- data.frame(

time_period = c("60s", "70s", "80s", "90s", "00s", "10s"),

actual_mean = c(0.02, 0.03, 0.04, 0.05, 0.08, 0.10), # Observed mean road density

sample_mean = c(0.01, 0.015, 0.02, 0.025, 0.03, 0.05), # Theoretical mean road density

lower_bound = c(0.007, 0.009, 0.012, 0.020, 0.028, 0.042), # 5th percentile

upper_bound = c(0.015, 0.018, 0.026, 0.031, 0.036, 0.06) # 95th percentile

)

# Convert time periods to a factor to ensure correct order

data_60km$time_period <- factor(data_60km$time_period, levels = c("60s", "70s", "80s", "90s", "00s", "10s"))

# Create the plot

p <- ggplot(data_60km, aes(x = time_period)) +

# Add a line for the actual mean

geom_line(aes(y = actual_mean, group = 1), color = "blue", size = 1) +

geom_point(aes(y = actual_mean), color = "purple", size = 3) +

# Add points for the sample mean, colored yellow

geom_point(aes(y = sample_mean), color = "yellow", size = 3) +

geom_errorbar(aes(ymin = lower_bound, ymax = upper_bound), color = "red", width = 0.2) +

# Add title and labels

labs(x = "Time Period",

y = "Mean") +

ylim(0, 0.12) +

# Customize theme

theme(

panel.background = element_rect(fill = NA),

panel.grid.major = element_blank(),

panel.grid.minor = element_blank(),

axis.ticks.length = unit(2, "mm"),

axis.line = element_line(colour = "black", size = 0.5),

axis.title.y = element_text(angle = 90, vjust = 0.5, hjust = 1),

axis.text.y = element_text(angle = 90, vjust = 0.5, margin = margin(r = 45, unit = "pt")),

axis.title.x = element_text(margin = margin(t = 45, unit = "pt")),

axis.line.x = element_line(arrow = arrow(type = "closed", length = unit(0.01, "mm"))),

axis.line.y = element_line(arrow = arrow(type = "closed", length = unit(0.01, "mm")))

)

# Display the plot

print(p)

############ The code for random forest model with spatial filters##############

###################Spatial autocorrelation calculation######################

library(vegan)

library(ncf) # correlog function

library(mgcv)

library(relaimpo)

library(sp) ### for calculate distance

library(spdep)

library(randomforest)

######################example data###############################

FID lon lat disroad pre meanhigh max_minh people ndvi

1 -76.47209276 -26.35090632 3.79621430802997 800.840255029733 3.26085996149143 86.2240823567845 3.57289223234814 0.105041741859168

2 103.78984875977 -24.04053991 3.01260281780257 792.008991246761 3.67853259942951 111.616730422247 3.92918224930488 0.402505600824952

3 -32.76830815 -38.32197648 7.05357011214981 996.403951884667 3.40093414213672 171.676251804456 3.80228290564002 0.455676587298512

4 137.886265441775 -75.60487578 6.50212260521265 973.564973428653 3.54468313598479 106.871183891781 3.91723076013212 0.624645246425644

5 158.568222345784 -24.21823148 1.9816669253426 490.131445049914 3.23905149945933 117.042534577195 3.56187109663231 0.078855280065909

6 -163.5996602 -57.95751337 4.80970509830143 1208.11469114388 3.56072305738736 135.576167376712 3.35598127658247 0.341982724610716

7 10.1179756969213 6.48966971319169 3.208104354 1049.94514719704 3.59536080254796 69.6818553959019 3.95412619573695 0.0236862907186151

8 141.270855981857 0.710768164135516 0.858497858916072 1483.24147462967 3.47461215606095 51.9527552765794 3.03123270612285 0.341516438405961

9 18.5166052076966 80.1063186256215 5.30024026237629 1137.03964761022 3.65078090594093 151.650459121447 3.78689941731337 0.783361223293468

10 -15.61869529 -28.56216889 4.84157658148282 910.608138207602 3.63622220117371 175.273341638967 3.98712760733899 0.741210231790319

11 164.460004325956 -6.351520652 4.80526146497384 1559.47822939162 3.69312830918029 190.439522173256 3.5343686914778 0.8161711092107

12 -16.79970377 -75.14438731 5.43230508360246 1566.4452047831 3.56435838726095 58.7675920804031 3.9936848595197 0.055563315981999

13 63.925428763032 64.8192322952673 6.76493032879317 756.257636741964 3.55284342749143 150.203869130928 3.12008190907916 0.151513537624851

14 26.1480247043073 -18.7810848 5.41119500896677 1093.80639119108 3.07209584078618 107.878481096122 3.52137189542434 0.653105947654694

15 -142.9471142 42.4618830671534 3.76712831425294 957.75061627357 3.24634733182088 136.419356591068 3.52361903245339 0.392788993660361

16 143.936989344656 -59.08618692 3.53040149615744 1037.4102293108 3.05321254476577 70.8221266744658 3.97926131723063 0.183773728553206

17 -91.40841563 -8.142906362 4.73639441391764 1045.50854571376 3.64234360449966 88.3750779903494 3.99196130065726 0.128402956528589

18 -164.8585679 48.6368566984311 5.6200339731579 747.61990742619 3.43101442260998 96.9591326196678 3.88135405378151 0.128896335605532

19 -61.94854106 -78.7230003 2.92063929459592 1057.11791604851 3.63030720723146 157.267671020236 3.5547698536436 0.624160754261538

20 163.621313693002 56.7146691167727 4.63138226231523 1349.84947262836 3.20591856792347 78.3019293332472 3.67030450352965 0.502002736320719

21 140.234153782949 -35.7943456 6.93453452033583 967.181999173039 3.41886061102965 71.327193477191 3.44935477853016 0.488161805551499

22 69.4092262163758 -24.35921496 4.78343981753103 967.414657860309 3.58995358990922 136.116742470767 3.89758735604219 0.872816388495266

23 50.5824529565871 -33.81969745 3.60315866481122 1279.71440126749 3.09290839396501 57.3725409107283 3.88581023710494 0.192297661909834

24 177.937119584531 -83.27340632 4.44810966314892 1179.67924813659 3.36574939881709 115.251147735398 3.7392678698934 0.759703308343887

25 56.0540876816958 3.38488621171564 7.22929709072396 670.301036323743 3.6135306253465 122.406524221878 3.84723300926121 0.219401078531519

26 75.0709685403854 32.2224148269743 6.10008792242827 1045.71139465244 3.63966947003693 119.7225351 3.89054201988702 0.156652296893299

27 15.863768896088 72.5820408482105 7.47335160016321 1330.70944693555 3.65150345135754 122.930290561635 3.90117303571352 0.144099411088973

28 33.8911273609847 -85.40519428 5.27819571432354 1283.05526998383 3.68234074527436 160.637732746545 3.80932558391945 0.886355442926288

29 -75.90249457 88.0340887326747 5.82055019298085 1083.99032076285 3.40411893828595 198.647946771234 3.65462328787357 0.668717395979911

30 -127.039087 -35.48022757 3.88308617530373 1144.24416142258 3.68146989350388 176.061253808439 3.84754293347355 0.456800217041746

31 166.68872371316 79.0449121082202 6.21074133786067 760.612957562263 3.5816559926966 66.7889483156614 3.94560989514822 0.716003178153187

32 144.827656242996 33.7693647108972 3.98733291562152 1060.02631337627 3.42941723957392 179.213281418197 3.69263772556123 0.707156684715301

33 68.6539002321661 -9.533340926 2.15886899060851 809.110212962436 3.40913213009862 135.17769316677 3.31895754047066 0.646718055708334

34 106.368270367384 56.9660649821162 5.25598593136825 908.396386380611 3.26535256863511 152.491837984417 3.81760372300412 0.915973308961838

35 -171.1390736 -82.89014439 8.89170243546287 1187.12073675592 3.60313578668896 168.440821813419 3.72628525532839 0.199645065004006

36 -7.993450407 43.0257033929229 6.60182867914612 772.621377661771 3.44409344072651 110.378829983529 3.96918462189787 0.434992756228894

37 93.0454335082322 -27.23049113 7.33050677989252 1053.38365017461 3.44920977398319 177.991578413639 3.50051834077407 0.0370358994696289

38 -102.0931431 59.2651482252404 5.7177114461863 1085.66640789431 3.12483126924271 78.4408298670314 3.90245622464292 0.157417114591226

39 -65.45483725 6.39745050575584 3.78288564336755 1010.98239396793 3.39105445371808 124.358244962059 3.89030312254519 0.702247529989108

40 -96.61471727 -40.58183455 4.59551828977943 1364.43776436708 3.66084761498309 68.0066523840651 3.85054465251619 0.740078432252631

41 -128.5919919 54.1706781461835 4.45350378637615 795.53053428495 3.50211991650522 84.2994959326461 3.77564504275725 0.429731045616791 -0.002369588 0.00162737104604392 -0.003229854

42 -30.76331911 -73.50395494 4.06260044043583 1121.22605228712 3.05049895454247 185.824684472755 3.80487137390095 0.350933959940448

43 -31.05924253 59.7793993586674 6.40833456785943 982.213886509866 3.40717537511558 121.066230977885 3.94438090870347 0.484288793755695

44 -47.21563767 -40.16608695 2.60527299526918 947.833551233215 3.1624392327954 131.071516883094 3.73703881583421 0.023608157876879

45 -125.1198908 45.5598179250956 6.73273226422555 1092.81824677048 3.32408025257648 196.069297846407 3.99220261267037 0.848993916762993

46 -130.0298172 83.5474626207724 6.72830497245179 795.919881863214 3.58861668809388 195.864353224169 3.21068554434113

47 -96.1077242 -75.3360192 2.6027552818625 737.309816696319 3.39865179655393 152.803920442238 3.77839443608235 0.185790497576818

48 -12.25351791 63.7856543669477 6.27898399580107 901.103824903315 3.54873816553976 166.993872972671 3.43206478118074 0.547376699512824

49 -84.24984947 54.4028790760785 9.86045330468902 1350.35142995109 3.47523393874551 53.067982615903 3.56444321924234 0.87369135953486

50 128.817977523431 -20.66875117 3.88556903611678 1011.1529549225 3.62633480580339 159.26986108534 3.93293202294536 0.229505306575447

# Construct the weight matrix i.sw1000 and calculate Moran's I index for the dependent variable

i.nb1000 <- dnearneigh(as.matrix(data[, 1:2]), 0, 300, longlat = TRUE)

i.sw1000 <- nb2listw(i.nb1000, glist = NULL, style = "W", zero.policy = TRUE)

moran.test(data[, 3], i.sw1000, zero.policy = TRUE)

# Calculate the first 40 spatial filters using the eigs method from the "RSpectra" package ###############

#### Calculating distance matrix ####

dis_coun <- spDists(as.matrix(data[, 1:2]), longlat = TRUE)

#### If distance > 300, replace it with 1200

dis_coun[which(dis_coun > 300)] <- 1200 # Distance matrix

#### Principal Coordinates of Neighbourhood Matrix

library(RSpectra)

# Used to compute the top n important vectors to reduce computation, referencing Multi-scaled drivers of severity patterns vary across land

# ownerships for the 2013 Rim Fire, California

eigens <- eigs(

dis_coun,

40,

which = "LM"

)

plot(eigens$values, type = "l", xlab = "Rank of eigenvalues", ylab = "Eigenvalues")

# According to the rules for adding spatial filters, select the minimum number of meaningful filters until the spatial autocorrelation of the random forest model residuals disappears

# Finally selected PCNM11, PCNM36, PCNM38

data$PCNM11 <- eigens$vectors[, 11]

data$PCNM36 <- eigens$vectors[, 36]

data$PCNM38 <- eigens$vectors[, 38]

#########################example data###############################

#################Examples of datasets after adding three spatial filters#########

FID lon lat disroad pre meanhigh max_minh people ndvi PCNM11 PCNM36 PCNM38

1 -76.47209276 -26.35090632 3.79621430802997 800.840255029733 3.26085996149143 86.2240823567845 3.57289223234814 0.105041741859168 -0.002369588 0.00162737104604395 -0.003229854

2 103.78984875977 -24.04053991 3.01260281780257 792.008991246761 3.67853259942951 111.616730422247 3.92918224930488 0.402505600824952 0.0176617161768096 0.00352009635439952 -0.004964987

3 -32.76830815 -38.32197648 7.05357011214981 996.403951884667 3.40093414213672 171.676251804456 3.80228290564002 0.455676587298512 -0.002369588 0.00162737104604407 -0.003229854

4 137.886265441775 -75.60487578 6.50212260521265 973.564973428653 3.54468313598479 106.871183891781 3.91723076013212 0.624645246425644 0.000140894717687493 0.0403345457696189 -0.006182343

5 158.568222345784 -24.21823148 1.9816669253426 490.131445049914 3.23905149945933 117.042534577195 3.56187109663231 0.078855280065909 -0.002369588 0.00162737104604421 -0.003229854

6 -163.5996602 -57.95751337 4.80970509830143 1208.11469114388 3.56072305738736 135.576167376712 3.35598127658247 0.341982724610716 -0.002369588 0.0016273710460441 -0.003229854

7 10.1179756969213 6.48966971319169 3.208104354 1049.94514719704 3.59536080254796 69.6818553959019 3.95412619573695 0.0236862907186151 -0.002369588 0.00162737104604412 -0.003229854

8 141.270855981857 0.710768164135516 0.858497858916072 1483.24147462967 3.47461215606095 51.9527552765794 3.03123270612285 0.341516438405961 0.240403281418314 0.00312210556008553 -0.004688901

9 18.5166052076966 80.1063186256215 5.30024026237629 1137.03964761022 3.65078090594093 151.650459121447 3.78689941731337 0.783361223293468 -0.002369588 0.00162737104604411 -0.003229854

10 -15.61869529 -28.56216889 4.84157658148282 910.608138207602 3.63622220117371 175.273341638967 3.98712760733899 0.741210231790319 -0.002369588 0.001627371046044 -0.003229854

11 164.460004325956 -6.351520652 4.80526146497384 1559.47822939162 3.69312830918029 190.439522173256 3.5343686914778 0.8161711092107 -0.002369588 0.00162737104604402 -0.003229854

12 -16.79970377 -75.14438731 5.43230508360246 1566.4452047831 3.56435838726095 58.7675920804031 3.9936848595197 0.055563315981999 -0.002369588 0.00162737104604396 -0.003229854

13 63.925428763032 64.8192322952673 6.76493032879317 756.257636741964 3.55284342749143 150.203869130928 3.12008190907916 0.151513537624851 -0.021997165 0.00282047821388783 -0.004454604

14 26.1480247043073 -18.7810848 5.41119500896677 1093.80639119108 3.07209584078618 107.878481096122 3.52137189542434 0.653105947654694 -0.002369588 0.00162737104604383 -0.003229854

15 -142.9471142 42.4618830671534 3.76712831425294 957.75061627357 3.24634733182088 136.419356591068 3.52361903245339 0.392788993660361 0.00267072405835541 0.068822528558331 -0.008868858

16 143.936989344656 -59.08618692 3.53040149615744 1037.4102293108 3.05321254476577 70.8221266744658 3.97926131723063 0.183773728553206 -0.002369588 0.00162737104604372 -0.003229854

17 -91.40841563 -8.142906362 4.73639441391764 1045.50854571376 3.64234360449966 88.3750779903494 3.99196130065726 0.128402956528589 -0.002369588 0.00162737104604372 -0.003229854

18 -164.8585679 48.6368566984311 5.6200339731579 747.61990742619 3.43101442260998 96.9591326196678 3.88135405378151 0.128896335605532 -0.002369588 0.00162737104604364 -0.003229854

19 -61.94854106 -78.7230003 2.92063929459592 1057.11791604851 3.63030720723146 157.267671020236 3.5547698536436 0.624160754261538 0.00342152902125626 0.00424918600031004 -0.00504726

20 163.621313693002 56.7146691167727 4.63138226231523 1349.84947262836 3.20591856792347 78.3019293332472 3.67030450352965 0.502002736320719 -0.002369588 0.00162737104604381 -0.003229854

21 140.234153782949 -35.7943456 6.93453452033583 967.181999173039 3.41886061102965 71.327193477191 3.44935477853016 0.488161805551499 -0.002369588 0.00162737104604383 -0.003229854

22 69.4092262163758 -24.35921496 4.78343981753103 967.414657860309 3.58995358990922 136.116742470767 3.89758735604219 0.872816388495266 -0.002369588 0.00162737104604382 -0.003229854

23 50.5824529565871 -33.81969745 3.60315866481122 1279.71440126749 3.09290839396501 57.3725409107283 3.88581023710494 0.192297661909834 -0.002369588 0.00162737104604396 -0.003229854

24 177.937119584531 -83.27340632 4.44810966314892 1179.67924813659 3.36574939881709 115.251147735398 3.7392678698934 0.759703308343887 0.000414655392701301 0.0328930492163202 -0.006400463

25 56.0540876816958 3.38488621171564 7.22929709072396 670.301036323743 3.6135306253465 122.406524221878 3.84723300926121 0.219401078531519 -0.002369588 0.0016273710460439 -0.003229854

26 75.0709685403854 32.2224148269743 6.10008792242827 1045.71139465244 3.63966947003693 119.7225351 3.89054201988702 0.156652296893299 -0.002369588 0.00162737104604386 -0.003229854

27 15.863768896088 72.5820408482105 7.47335160016321 1330.70944693555 3.65150345135754 122.930290561635 3.90117303571352 0.144099411088973 -0.035252616 0.00291757945994256 -0.004532627

28 33.8911273609847 -85.40519428 5.27819571432354 1283.05526998383 3.68234074527436 160.637732746545 3.80932558391945 0.886355442926288 0.000764409357964719 -0.003182915 0.462568655213147

29 -75.90249457 88.0340887326747 5.82055019298085 1083.99032076285 3.40411893828595 198.647946771234 3.65462328787357 0.668717395979911 0.00804258866729129 0.00251150407244391 -0.000304478

30 -127.039087 -35.48022757 3.88308617530373 1144.24416142258 3.68146989350388 176.061253808439 3.84754293347355 0.456800217041746 -0.002369588 0.00162737104604398 -0.003229854

31 166.68872371316 79.0449121082202 6.21074133786067 760.612957562263 3.5816559926966 66.7889483156614 3.94560989514822 0.716003178153187 -0.002369588 0.00162737104604397 -0.003229854

32 144.827656242996 33.7693647108972 3.98733291562152 1060.02631337627 3.42941723957392 179.213281418197 3.69263772556123 0.707156684715301 -0.002369588 0.0016273710460439 -0.003229854

33 68.6539002321661 -9.533340926 2.15886899060851 809.110212962436 3.40913213009862 135.17769316677 3.31895754047066 0.646718055708334 -0.002369588 0.00162737104604393 -0.003229854

34 106.368270367384 56.9660649821162 5.25598593136825 908.396386380611 3.26535256863511 152.491837984417 3.81760372300412 0.915973308961838 -0.002369588 0.00162737104604381 -0.003229854

35 -171.1390736 -82.89014439 8.89170243546287 1187.12073675592 3.60313578668896 168.440821813419 3.72628525532839 0.199645065004006 0.00274446005333926 0.065007116014711 -0.010142712

36 -7.993450407 43.0257033929229 6.60182867914612 772.621377661771 3.44409344072651 110.378829983529 3.96918462189787 0.434992756228894 -0.002369588 0.00162737104604399 -0.003229854

37 93.0454335082322 -27.23049113 7.33050677989252 1053.38365017461 3.44920977398319 177.991578413639 3.50051834077407 0.0370358994696289 -0.002369588 0.00162737104604395 -0.003229854

38 -102.0931431 59.2651482252404 5.7177114461863 1085.66640789431 3.12483126924271 78.4408298670314 3.90245622464292 0.157417114591226 -0.002369588 0.00162737104604378 -0.003229854

39 -65.45483725 6.39745050575584 3.78288564336755 1010.98239396793 3.39105445371808 124.358244962059 3.89030312254519 0.702247529989108 0.569193406460225 0.00310602084470995 -0.004676989

40 -96.61471727 -40.58183455 4.59551828977943 1364.43776436708 3.66084761498309 68.0066523840651 3.85054465251619 0.740078432252631 -0.002369588 0.00162737104604395 -0.003229854

41 -128.5919919 54.1706781461835 4.45350378637615 795.53053428495 3.50211991650522 84.2994959326461 3.77564504275725 0.429731045616791 -0.002369588 0.00162737104604392 -0.003229854

42 -30.76331911 -73.50395494 4.06260044043583 1121.22605228712 3.05049895454247 185.824684472755 3.80487137390095 0.350933959940448 -0.002369588 0.00162737104604405 -0.003229854

43 -31.05924253 59.7793993586674 6.40833456785943 982.213886509866 3.40717537511558 121.066230977885 3.94438090870347 0.484288793755695 -0.002369588 0.00162737104604402 -0.003229854

44 -47.21563767 -40.16608695 2.60527299526918 947.833551233215 3.1624392327954 131.071516883094 3.73703881583421 0.023608157876879 -0.02089049 0.00280731501486342 -0.004443827

45 -125.1198908 45.5598179250956 6.73273226422555 1092.81824677048 3.32408025257648 196.069297846407 3.99220261267037 0.848993916762993 -0.002369588 0.00162737104604404 -0.003229854

46 -130.0298172 83.5474626207724 6.72830497245179 795.919881863214 3.58861668809388 195.864353224169 3.21068554434113 0.652646586764604 -0.00039307 -0.2396304 -0.006089828

47 -96.1077242 -75.3360192 2.6027552818625 737.309816696319 3.39865179655393 152.803920442238 3.77839443608235 0.185790497576818 -0.002369588 0.00162737104604419 -0.003229854

48 -12.25351791 63.7856543669477 6.27898399580107 901.103824903315 3.54873816553976 166.993872972671 3.43206478118074 0.547376699512824 -0.026906497 0.0028667757496063 -0.004492126

49 -84.24984947 54.4028790760785 9.86045330468902 1350.35142995109 3.47523393874551 53.067982615903 3.56444321924234 0.87369135953486 -0.002369588 0.00162737104604423 -0.003229854

50 128.817977523431 -20.66875117 3.88556903611678 1011.1529549225 3.62633480580339 159.26986108534 3.93293202294536 0.229505306575447 -0.002369588 0.00162737104604416 -0.00322985

# Random forest model with spatial filters

set.seed(123)

train_index <- sample(1:nrow(data), 0.75 * nrow(data))

train_data <- data[train_index, ]

test_data <- data[-train_index, ]

rf_model_Pcnm <- randomForest(distance ~ pre + meanhigh + max_minh + people + ndvi + PCNM11 + PCNM36 + PCNM38, data = train_data, ntree = 700, mtry = 2, importance = TRUE)

# Residual extraction

res_model_PCNM <- train_data$distance - rf_model_Pcnm$predicted

# Test for the presence of spatial autocorrelation in residuals

i.nb1000 <- dnearneigh(as.matrix(train_data[, 1:2]), 0, 300, longlat = TRUE)

i.sw1000 <- nb2listw(i.nb1000, glist = NULL, style = "W", zero.policy = TRUE)

moran.test(res_model_PCNM, i.sw1000, zero.policy = TRUE)

# Random forest model without spatial filters

set.seed(123)

rf_model_Nocnm <- randomForest(distance ~ pre + meanhigh + max_minh + people + ndvi, data = train_data, ntree = 700, mtry = 2, importance = TRUE)

# Residual extraction

res_model_NoPCNM <- train_data$distance - rf_model_Nocnm$predicted

# Test for the presence of spatial autocorrelation in residuals

i.nb1000 <- dnearneigh(as.matrix(train_data[, 1:2]), 0, 300, longlat = TRUE)

i.sw1000 <- nb2listw(i.nb1000, glist = NULL, style = "W", zero.policy = TRUE)

moran.test(res_model_NoPCNM, i.sw1000, zero.policy = TRUE)

# Changes in Moran's I index before and after adding spatial filters

library(ncf)

library(corrgram)

Moran.cor.sr1 <- correlog(data[, 1], data[, 2], data[, 3], increment = 200, resamp = 100, latlon = TRUE) # Moran's I threshold plot for the response variable

Moran.cor.srNopcnm <- correlog(data[, 1], data[, 2], res_model_NoPCNM, increment = 200, resamp = 100, latlon = TRUE)

Moran.cor.srPcnm <- correlog(data[, 1], data[, 2], res_model_PCNM, increment = 200, resamp = 100, latlon = TRUE)

######### Hyperparameter tuning for the random forest model ########

# Load necessary packages

library(randomForest)

library(caret)

# Use trainControl function to set up cross-validation. For example, we can use 5-fold cross-validation (k = 5).

# Set cross-validation parameters

train_control <- trainControl(

method = "cv", # Use cross-validation

number = 5, # 5-fold cross-validation

verboseIter = TRUE, # Print results for each iteration

allowParallel = TRUE # Allow parallel computation (if possible)

)

# Step 3: Define hyperparameter grid

# Define candidate ranges for mtry and ntree. mtry is the number of variables randomly selected at each split, and ntree is the number of trees in the forest.

# Define hyperparameter grid

tune_grid <- expand.grid(

mtry = c(2, 4, 6, 8) # Candidate values for mtry

)

# Train the random forest model

rf_model <- train(

distance ~ pre + meanhigh + max_minh + people + ndvi + PCNM11 + PCNM36 + PCNM38,

data = train_data,

method = "rf", # Random forest method

trControl = train_control,

tuneGrid = tune_grid,

importance = TRUE # Enable feature importance calculation

)

# View the best hyperparameters

print(rf_model$bestTune)

# View model performance summary

print(rf_model)

# Get predictions

predictions <- predict(rf_model, newdata = test_data)

# Calculate Mean Squared Error (MSE)

mse <- mean((test_data$distance - predictions)^2)

cat("Mean Squared Error:", mse, "\n")

# Calculate R-squared (variance explained)

r_squared <- cor(test_data$distance, predictions)^2

cat("R-squared:", r_squared, "\n")

# Plot feature importance

varImpPlot(rf_model$finalModel)

# Test random forest model performance

set.seed(123)

train_index <- sample(1:nrow(data), 0.75 * nrow(data))

train_data <- data[train_index, ]

test_data <- data[-train_index, ]

set.seed(123)

rf_model_train <- randomForest(distance ~ pre + meanhigh + max_minh + people + ndvi + PCNM11 + PCNM36 + PCNM38, data = train_data, ntree = 1000, mtry = 3, importance = TRUE)

rf_model_train

trainpred <- predict(rf_model_train, newdata = train_data)

defaultSummary(data.frame(obs = train_data$distance, pred = trainpred))

testpred <- predict(rf_model_train, newdata = test_data)

defaultSummary(data.frame(obs = test_data$distance, pred = testpred))

#### Calculate relative importance of each variable ##########

# Calculate relative importance of variables

# Get feature importance

feature_importance <- importance(rf_model_Pcnm)

# Print the importance percentage of each explanatory variable

print(feature_importance)

# Convert feature importance to percentage form

total_importance <- sum(feature_importance[,"%IncMSE"]) # Calculate total importance value

feature_importance_pct <- feature_importance[,"%IncMSE"] / total_importance * 100 # Calculate the percentage of importance for each explanatory variable

# Print the importance percentage of each explanatory variable

print(feature_importance_pct)
